# Synthetic Extracellular Matrix of Polyvinyl Alcohol Nanofibers for Three-Dimensional Cell Culture

**DOI:** 10.3390/jfb15090262

**Published:** 2024-09-10

**Authors:** Thi Xuan Thuy Tran, Gyu-Min Sun, Hue Vy An Tran, Young Hun Jeong, Petr Slama, Young-Chae Chang, In-Jeong Lee, Jong-Young Kwak

**Affiliations:** 1 Department of Pharmacology, School of Medicine, Ajou University, Suwon 16499, Republic of Korea; ttxt@ajou.ac.kr (T.X.T.T.); rbals50029490@gmail.com (G.-M.S.); thvyan@gmail.com (H.V.A.T.); 2 Department of Medical Sciences, The Graduate School, Ajou University, Suwon 16499, Republic of Korea; 3 School of Mechanical Engineering, Kyungpook National University, Daegu 41566, Republic of Korea; yhjeong@knu.ac.kr; 4 Department of Animal Morphology, Physiology and Genetics, Faculty of AgriSciences, Mendel University in Brno, Zemedelska 1, 613 00 Brno, Czech Republic; petr.slama@mendelu.cz; 5 Department of Cell Biology, School of Medicine, Catholic University of Daegu, Daegu 42272, Republic of Korea; ycchang@cu.ac.kr; 6 3D Immune System Imaging Core Center, Ajou University, Suwon 16499, Republic of Korea

**Keywords:** three-dimensional cell cultures, polyvinyl alcohol, nanofibers, extracellular matrix

## Abstract

An ideal extracellular matrix (ECM) replacement scaffold in a three-dimensional cell (3D) culture should induce in vivo-like interactions between the ECM and cultured cells. Highly hydrophilic polyvinyl alcohol (PVA) nanofibers disintegrate upon contact with water, resulting in the loss of their fibrous morphology in cell cultures. This can be resolved by using chemical crosslinkers and post-crosslinking. A crosslinked, water-stable, porous, and optically transparent PVA nanofibrous membrane (NM) supports the 3D growth of various cell types. The binding of cells attached to the porous PVA NM is low, resulting in the aggregation of cultured cells in prolonged cultures. PVA NMs containing integrin-binding peptides of fibronectin and laminin were produced to retain the blended peptides as cell-binding substrates. These peptide-blended PVA NMs promote peptide-specific cell adherence and growth. Various cells, including epithelial cells, cultured on these PVA NMs form layers instead of cell aggregates and spheroids, and their growth patterns are similar to those of the cells cultured on an ECM-coated PVA NM. The peptide-retained PVA NMs are non-stimulatory to dendritic cells cultured on the membranes. These peptide-retaining PVA NMs can be used as an ECM replacement matrix by providing in vivo-like interactions between the matrix and cultured cells.

## 1. Introduction

The growth and differentiation of cells in living tissues are affected by the extracellular matrix (ECM) in a three-dimensional (3D) environment. Cells interface with the ECM through various spatially segregated adhesive structures. The ECM comprises a basement membrane and an interstitial matrix, in which the significant proteins are collagen and cell-adhesive proteins, such as fibronectin and laminin, which connect the cells with collagen fibers in the ECM [1]. Cells interact with fibronectin and laminin through integrin receptors on the cell surface [2]. Integrins binding to fibronectin and laminin proteins affect cell adhesion, behavior, and functions [3]. Cell culture, a fundamental technique in biological research and biotechnology, provides a platform for studying cell behavior, physiology, and responses to various stimuli. Traditional two-dimensional (2D) cell culture does not fully mimic the complex 3D microenvironment of living tissues. Hence, biomimetic 3D cell culture methods that can improve cell culture efficiency have been developed [4,5]. For such methods, an ideal matrix for ECM replacement in 3D cell culture should provide in vivo-like ECM protein–cell interactions.

The nanofibrous scaffold structure should mimic the configuration of the native ECM in biological tissues, providing a 3D architecture for cell culture [6,7]. Various fabrication techniques have been used to produce 3D porous scaffolds for tissue-engineering applications [8,9]. For example, electrospinning is a unique technology that can produce nonwoven fibrous structures with fiber diameters ranging from nanometers to microns [10,11]. Many biocompatible and biodegradable synthetic polymers, such as polyvinyl alcohol (PVA), polycaprolactone, poly(lactic acid), and poly(lactic-*co*-glycolic acid), have been directly electrospun into nanofibers for cell culture, tissue engineering, and drug delivery applications [12,13,14,15]. The chemical, mechanical, and physical characteristics of nanofibers may affect cellular functions, such as the adherence, growth, proliferation, and differentiation of cells cultured on a nanofiber scaffold [16,17]. However, nanofiber scaffolds differ from natural ECMs because they lack specific biological motifs that interact with cultured cells and modify cell function. This problem has been addressed by surface functionalization using ECMs, proteins, and peptides incorporating chitosan and keratin and blending with gelatin [18,19,20,21,22,23,24,25,26].

PVA is a semicrystalline, hydrophilic, biocompatible, and non-toxic polymer [27,28]. PVA polymers are widely used as biomaterials because of their high hydrophilicity [29]. However, highly hydrophilic PVA nanofibers disintegrate upon contact with water, resulting in the loss of their fibrous morphology in cell cultures [30]. Therefore, chemical crosslinking is carried out by introducing crosslinkers, such as glutaraldehyde (GA), hexamethyl diisocyanate, and maleic acid, which create irreversible covalent bonds between polymer chains [28,31,32,33]. Radiation and heat treatment may also induce the physical crosslinking of PVA nanofibers [34]. The pure form of PVA exhibits intrinsically low adhesion to proteins and is non-adhesive to cells, and synthetic PVA nanofibers lack cell-binding substrates [19,35]. Thus, the use of crosslinked PVA nanofibers as a matrix for cell culture is limited. Moreover, the non-transparency of the insoluble nanofiber matrix is not suitable for observing cells in 3D cell cultures.

A tissue-microenvironment-like matrix should have structural and specific adhesive characteristics in the ECM to maintain cellular functions. In this study, we attempted to address this issue by producing water-stable and optically transparent PVA nanofibers by increasing the crosslinking properties of the nanofibers, followed by the production of receptor-specific peptide-retained nanofibers to enhance the binding capacity of cultured cells to the PVA nanofiber matrix and maintain the 3D growth of cells as in cell–ECM interactions. The fabricated water-stable and optically transparent PVA nanofibers may prove invaluable in 3D cell culture, thus facilitating the study of cell behavior, disease mechanisms, drug responses, and tissue development.

## 2. Materials and Methods

### 2.1. Materials

PVA, poly(acrylic acid) (PAA), ethanol, GA, hydrochloric acid (HCl), dimethyl sulfoxide (DMSO), lipopolysaccharide (LPS) from *Escherichia coli* (O111:b4), sorafenib, and fibronectin were obtained from Sigma-Aldrich (St Louis, MO, USA) and used as received. Antibodies against CD11c and CD86 and 7-amino actinomycin D (7-AAD) were purchased from eBioscience (San Diego, CA, USA). Dulbecco’s modified Eagle’s medium (DMEM), RPMI-1640 medium, fetal bovine serum (FBS), penicillin/streptomycin, glutamine, trypsin-ethylenediaminetetraacetic acid (0.05%), and laminin were purchased from Gibco (Rockville, MD, USA). Phalloidin-ifluor 488 was purchased from Abcam (Cambridge, UK). Hoechst 33342, CellTracker dyes, anti-zonal occludin (ZO)-1 antibody, and Alexa Fluor 488- and 594-conjugated secondary antibodies were obtained from Thermo Fischer Scientific (Waltham, MA, USA). Arg-Gly-Asp (RGD), Tyr-Ile-Gly-Ser-Arg (YIGSR), Ile-Lys-Val-Ala-Val (IKVAV), and other peptides were purchased from Peptron (Daejeon, Republic of Korea).

### 2.2. Electrospinning

The PVA nanofibers were prepared according to a previously published procedure [36]. PVA (99% hydrolyzed, *M*_w_ = 89,000–98,000) was dissolved in distilled water at 85 °C overnight with stirring to obtain a clear PVA solution (10% *w*/*v*). To prepare PVA nanofibers mixed with PAA (PVA/PAA nanofibers), PAA (*M*_w_ = 2000) (0.2% *w*/*v*) was added. To fabricate peptide-blended nanofibers, peptides (100 μg/mL) were added to the PVA/PAA solution and dissolved at 85 °C for 12 h using a heating magnetic stirrer. The solution was cooled to 25 °C, and the GA solution was mixed with solutions of PVA/PAA in a final concentration of 2%. Solutions were prepared by continuously stirring the mixture for 30 min at room temperature until they became clear and homogenous. The nanofibers were fabricated via electrospinning (NanoNC, Seoul, Republic of Korea). Using a syringe pump, the average flow rate was approximately 6 μL/min. Nanofibers were collected on a grounded aluminum foil collector at 100 rpm under 25 °C and 50% humidity for 5 h. The nozzle tip-to-collector distance was 14 cm with an electrical potential of approximately 12 kV. After fabrication, the nanofibers were heat-treated at 60 °C for 40 s.

### 2.3. Post-Crosslinking of Nanofibers with HCl Vapor

The PVA/PAA/GA nanofibers were quickly exposed to HCl vapor after electrospinning because of their high hydrophilic properties under moist conditions. A Petri dish containing a HCl solution (2 mL) was placed at the bottom of the desiccator. The electrospun PVA/PAA/GA nanofiber membranes (NMs) were then exposed to the HCl vapor for 2 min. The HCl vapor-treated membranes were kept in a vacuum chamber before being used for cell culture.

### 2.4. Assay of Nanofiber Morphology

To verify the water stability of the PVA nanofibers, the PVA NMs were soaked in distilled water and dried, and scanning electron microscope (SEM) images were obtained. Nanofibers on carbon tape (SPI Supplies Inc., Seoul, Republic of Korea) were coated with a thin layer of gold using an autosputter coater. The morphology and fiber diameters, along with their distribution in the electrospun samples, were determined using an SEM (SNE-4500M, Sec Co., Suwon, Republic of Korea) with an acceleration voltage of 30 KV. The average diameter of the electrospun nanofibers was measured from the SEM micrographs at an original magnification of 10,000× using ImageJ software (version 1.54h).

### 2.5. Light Transmittance

The light transmittance of the PVA NM attached to a cuvette was measured with a spectrophotometer (Ultrospec 6300 Pro UV/Vis spectrophotometer, Amersham Biosciences Corp., Uppsala, Sweden) at 25 °C. The thickness of PVA nanofiber mats was 50 ± 7 μm, and an identical cuvette filled only with distilled water served as a reference.

### 2.6. Fourier Transform Infrared Spectra

PVA and peptide-blended PVA NMs were treated with or without HCl vapor for 2 min, followed by soaking in distilled water or 1 M sodium hydroxide (NaOH) for 12 h. (Fourier transform infrared spectra (FTIR) imaging measurements of the dried PVA NMs in each step were performed using an FTIR System (Nicolet iS50 FTIR spectrometer, Thermo Scientific Co., Seoul, Republic of Korea). The spectra were collected in the range of 4000–500 cm^−1^.

### 2.7. Water Contact Angle Measurements

The contact angles of the electrospun polycaprolactone, PVA, and peptide-blended PVA NMs (*n* = 3) were tested using a drop-size analyzer (Phoenix-MT model, SEO Co., Suwon, Republic of Korea). Briefly, a 10 μL droplet of distilled water was released onto each surface using a needle, and the change in contact angle over 20 s was measured.

### 2.8. Release Assay of Fluorescence from Florescence-Conjugated Peptide-Blended Nanofibers

The release of fluorescence was studied by incubating the fluorescence-conjugated peptide-blended PVA NM in distilled water and 1 M NaOH solution at 25 °C under continuous agitation (100 rpm). The supernatant was withdrawn, and the released fluorescence was measured using a fluorescence microplate reader (Synergy H1, BioTek Co., Incheon, Republic of Korea) at excitation and emission wavelengths of 490 and 520 nm for Fluorescein isothiocyanate (FITC) and 550 and 575 nm for carboxytetramethylrhodamine (TAMRA), respectively.

### 2.9. Cell Culture

MLE-12 mouse lung epithelial type II cells were purchased from the American Type Culture Collection (ATCC) (Manassas, VA, USA) and cultured in ATCC-formulated DMEM supplemented with 10% FBS, 1% penicillin/streptomycin, 20 mM N-2-hydroxyethylpiperazine-N-2-ethane sulfonic acid (HEPES), and 2 mM L-glutamine at 37 °C in 5% CO_2_. MLE-12 cells at passages 6–10 were used. NIH 3T3 mouse fibroblasts, CT-26 murine colon carcinoma cells, and HepG2 human hepatoma cells were obtained from the Korean Cell Bank (Seoul, Republic of Korea). They were maintained in DMEM media containing 10% FBS supplemented with 1% penicillin and streptomycin. The cells were subcultured every 3–4 days. Human primary colonic epithelial cells were obtained from Cell Biologics, Inc. (Chicago, IL, USA). The membranes attached to 8-well plates were sterilized by exposing them to UV light for 12 h and immersed in DMEM for 6 h to increase cell adhesion at 37 °C. Cells were fluorescence-stained by incubation with 5 mM CellTracker dyes for 30 min at 37 °C. Fluorescence-labeled and unlabeled cells (3 × 10^4^–1 × 10^5^ cells in 500 µL culture medium) were seeded on the membranes. Fibronectin- and laminin-coated PVA NMs were used in some experiments. In brief, the solution (120 μL) of fibronectin and laminin (10 μg/mL) was added to each PVA NM and then kept at 4 °C for 12 h. The protein-coated membrane was prewarmed for 1 h at 25 °C, and cells were seeded on them. After culturing for predetermined intervals, cell adhesion to the membrane was observed using confocal laser microscopy.

### 2.10. Culture of BMDCs on PVA NM

C57BL/6 mice were kept in accordance with the Institutional Animal Care and Use Committee of Ajou University (2018-0014). Bone marrow (BM) cells were harvested from the femurs and tibias of the mice, and BM-derived dendritic cells (BMDCs) were obtained from the BM precursors, as described previously [37]. Briefly, BM cells in a 6-well culture plate at a seeding density of 3 × 10^6^ cells/mL were differentiated in RPMI-1640 medium containing recombinant murine granulocyte macrophage-colony stimulating factor (GM-CSF) (20 ng/mL) and interleukin-4 (IL-4) (20 ng mL) (JW Creagene, Seoul, Republic of Korea). On the third day of incubation, the same concentration of GM-CSF and IL-4 was added, and half of the culture medium was refreshed. BMDCs were harvested on day 7 of incubation. By the immunomagnetic selection of CD11c^+^ cells, BMDCs were purified to >90% using CD11c Microbeads Ultrapure (Miltenyi Biotec, Bergisch-Gladbach, Germany). To investigate whether the BMDCs cultured on the PVA NM were activated, the cells were cultured on the PVA NM in an 8-well plate (2 × 10^5^ cells/mL) cultured with or without 1 µg/mL LPS for 24 h. At the end of the culture period, dead cells were excluded using the viability dye 7-AAD, and cell surface molecules on the 7-AAD-negative populations were determined by flow cytometry (MACSQuant VYB, Miltenyi Biotec). The flow cytometry data were analyzed using FlowJo v10 (Tree Star, Ashland, OR, USA). Cell-free supernatants were collected and stored at −80 °C to measure the secreted cytokine levels using standard enzyme-linked immunosorbent assay (ELISA) kits (R&D Systems).

### 2.11. Morphological Assay of Cultured Cells by SEM

For the morphological observation of the cells adhering to the PVA NM, the membrane was washed with phosphate-buffered saline (PBS) and then fixed with 3% GA in 0.1 M sodium phosphate buffer (pH = 7.2) for 1 h at 4 °C. After washing with PBS three times, the cells were treated with 1% OsO_4_ in the buffer for 1 h at 25 °C. The cells were washed three times with PBS and dehydrated using graded ethanol changes. The membranes were affixed to aluminum mounts with double-sided carbon tape and sputter-coated with gold. The cell structure was observed by an SEM.

### 2.12. Laser Confocal Microscopy

Fluorescence-labeled cells were cultured on PVA NMs and observed under a K1 confocal microscope (Nanoscope, Republic of Korea). Unlabeled cells cultured on culture plates and PVA NMs were fixed with 4% paraformaldehyde in PBS for 10 min and permeabilized with 0.2% Triton X-100 for 3 min at 25 °C. After washing with PBS three times, the cells were blocked using 5% goat serum in PBS for 1 h at 25 °C and incubated overnight at 4 °C with primary antibodies, including the anti-ZO-1 antibody (1:50 dilution) and the anti-CD86 antibody (1:100 dilution). The samples were washed with PBS three times and treated with the Alexa Fluor 594-conjugated secondary antibody (donkey anti-rabbit, 1:400 dilution) and phalloidin-iFluor 488 (1:1000 dilution) for 1 h. The cell nuclei were stained with Hoechst 33,342 in PBS (1:1000 dilution). IMARIS 9.3.1 (Bitplane, Belfast, Northern Ireland, UK) was used to create the 3D visualization of immunofluorescence images obtained by confocal microscopy. The 3D images were analyzed using the surface function of Imaris software (version 9.3.1).

### 2.13. Live Cell Imaging

Live imaging was performed using a ZEISS CellDiscoverer 7 with LSM900 at the 3D Immune System Imaging Core Facility of Ajou University. MLE-12 cells (5 × 10^4^ cells) were labeled with CellTracker Green (5 μM) for 30 min at 37 °C and seeded on a culture plate and PVA NM in a culture plate. Following the instruction manual, the cells were kept in the instrument for 24 h for live cell imaging.

### 2.14. Cell Proliferation Assay

Cell growth on the culture plate and PVA NM was assessed using the Cell Counting Kit (CCK)-8 assay, according to the manufacturer’s protocol (Dojindo Molecular Technologies, Gaithersburg, MD, USA). Briefly, cells were seeded at 3 × 10^4^ cells/500 μL on an 8-well plate and a PVA NM and cultured for five days at 37 °C under 5% CO_2_. For the cytotoxicity assay of HepG2 cells, the cells (5 × 10^4^ cells) were cultured on a culture plate and a PVA NM in various concentrations of sorafenib for three days. After discarding the culture medium, a medium and CCK-8 mixture (9:1) was added to the cells. After 30 min incubation at 37 °C, the absorbance at 450 nm was measured using a microplate reader (BioTek Co.). Cell viability was calculated as follows: (OD sample-OD blank/OD control-OD blank) × 100.

### 2.15. Statistical Analysis

The results are presented as mean ± standard deviation (SD). Student’s *t*-test was used to compare the means of paired or unpaired samples. A *p*-value of <0.05 was considered significant.

## 3. Results

### 3.1. Preparation of Water-Stable PVA Nanofibers

**Figure 1:**
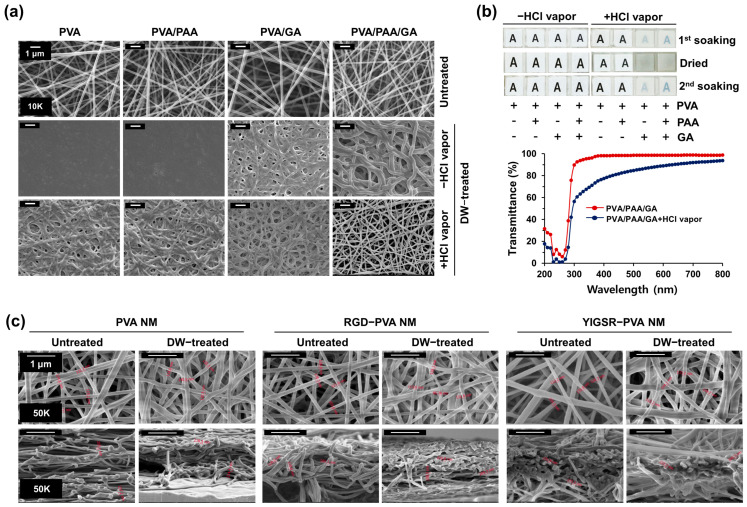
Production of water-stable and optically transparent PVA NMs. The membranes are untreated or soaked in distilled water and dried. (**a**) Structure of nanofibers measured by SEM. (**b**) Visual and optical transparency of membrane scanned using a spectrophotometer. (**c**) The structure and diameter of the PVA NMs measured by SEM.

PVA nanofibers were fabricated using an electrospinning method under various electrospinning conditions, including concentrations, flow rates, and tip-to-collector distances. After the initial experiments with PVA nanofibers, we chose the best condition of a 10% *w*/*v* solution of PVA and 0.2% *w*/*v* solution of PAA (hereafter referred to as PVA/PAA nanofibers). In a morphological analysis using an SEM, the fabrication of PVA/PAA nanofibers with 12 kV voltage, 6 µL/min flow rate, and distance of 14 cm shows the uniform distribution of nanofibers without any bead formation (Figure 1a). The diameter of the PVA/PAA nanofibers ranged from 160 nm to 220 nm (180 ± 25 nm). This method was adopted in the remainder of the experiments. When the PVA/PAA NM was soaked in distilled water to analyze the fiber stability during water contact, the membrane dissolved instantly in water and turned into a transparent gel-like material. The SEM image of the PVA/PAA NM shows a smooth membrane surface and the disappearance of the fiber structure after soaking the membrane in water. This result suggests that the degree of crosslinking is insufficient to stabilize the PVA nanofibers in water. A crosslinker, GA, was incorporated into the electrospinning solution to prevent the PVA/PAA nanofibers from dissolving in water (hereafter referred to as PVA/PAA/GA nanofibers). The electrospun PVA/PAA/GA nanofibers exhibited uniform fiber morphology. The PVA/PAA/GA nanofibers did not dissolve but were significantly swollen after treatment with water compared with the untreated control nanofibers. In comparison, the PVA/GA nanofibers fabricated without adding PAA dissolved in water because of improper crosslinking.

We performed further post-treatment to obtain more water-stable PVA nanofibers because post-electrospinning treatment with chemicals activates the crosslinking reactions of nanofibers. The stability of PVA-based coating films is affected by the presence of acidic additives and HCl vapor [38,39]. PVA/PAA/GA nanofibers were treated with HCl vapor for 2 min in a vacuum desiccator. The HCl vapor-treated PVA/PAA/GA nanofibers exhibited well-preserved fibrous and porous morphology even after water treatment. Analysis of the effect of the GA to PVA ratio in post-crosslinked PVA/PAA/GA nanofibrous membrane (hereafter referred to as the PVA NM) led to the determination of the optimal final concentration of GA in the electrospun solution as 2%. In comparison, adding GA alone to PVA is not sufficient for water stabilization after the post-crosslinking treatment, indicating that PAA also helps to maintain the morphology of the PVA nanofibers after contact with water.

### 3.2. Optical Transparency of Water-Stable PVA Nanofibers

Most NMs are optically non-transparent; hence, observation of the cultured cells on the membranes using an optical microscope is challenging. We tested the optical transparency of the PVA NM in distilled water. The PVA NM attached to an 8-well cell culture plate was placed on a printed letter to visually compare the transparency differences. The membrane was water-stable and optically transparent in water, as the letter “A” could be clearly seen, although it demonstrated decreased transparency compared to water-soluble PVA/PAA and PVA/GA NMs (Figure 1b). After water soaking, the dried PVA NM lost optical transparency but maintained reversible transparency during the second water treatment, suggesting that the transparency remained reversible. When the visible-light transmittance of the water-stable PVA NM was recorded using a spectrometer, the optical transparency was 80%. This result indicates that the post-crosslinked nanofibers retained fibrous structures with optical transparency during water contact.

### 3.3. Production of Peptide-Blended PVA Nanofibers

PVA nanofibers have no specific chemical or biological cell-binding characteristics. In this study, we fabricated nanofibers containing the cell-adhesive peptides of fibronectin and laminin because proteins quickly lose their activity upon chemical or physical processing. In fibronectin, the RGD peptide is an integrin recognition motif; in laminin, the representative cell binding site is the YIGSR sequence of the β1 chain [2]. The structure and diameter of the nanofibers in the RGD peptide-blended PVA NM (hereafter referred to as the RGD-PVA NM) and YIGSR peptide-blended PVA NM (hereafter referred to as the YIGSR-PVA NM) were similar to those in the PVA NM (Figure 1c). The average fiber diameter of PVA nanofibers before and after treatment with water on the surface measurement of each membrane using an SEM was 164.2 ± 13.3 nm and 184.1 ± 15.5 nm, respectively. In addition, the cross-sectional measurement of the membranes showed no significant change in fiber diameter between untreated and water-treated PVA NMs (205.6 ± 25.3 nm vs. 226.5 ± 27.5 nm). The diameters of the nanofibers in the RGD-PVA NM (187. 5 ± 20.3 nm) and YIGSR-PVA NM (170. 5 ± 26.7 nm) were similar to that in the PVA NM and were not significantly increased by water treatment. Moreover, the porosity of the water-treated PVA NM was not significantly increased compared to the untreated PVA NM (80.1 ± 4.5% vs. 77.9 ± 3.4%), and the water treatment of the RGD-PVA NM showed no changes in porosity compared to the untreated RGD-PVA NM (78.1 ± 2.7% vs. 82.0 ± 3.6%). Pro-His-Ser-Arg-Asn (PHSRN) is a synergy ligand of RGD in enhancing cell spread, and the IKVAV sequence from the α chain of laminin mediates cell attachment [40,41]. When PHSRN, IKVAV, scrambled peptides, such as Asp-Arg-Gly (DRG), and two different peptides with RGD and PHSRN were blended into the PVA nanofibers, we observed constant nanofiber diameters and water stability, with a slight increase in fiber diameter in the water-treated nanofibers (Figure S1). In addition, the optical transparency was not disturbed by the addition of peptides to the PVA nanofibers. Next, the contact angles of the peptide-blended PVA nanofibers were compared with those of the control PVA and polycaprolactone nanofibers. The contact angle of the PVA nanofibers was about 40°, but that of the polycaprolactone nanofibers was significantly higher (105°) (Figure S2). We observed no significant changes in wettability when the PVA nanofibers were blended with RGD and YIGSR, indicating that the blending of peptide did not alter the hydrophilicity of the PVA NM.

### 3.4. Fourier Transform Infrared Spectra

**Figure 2:**
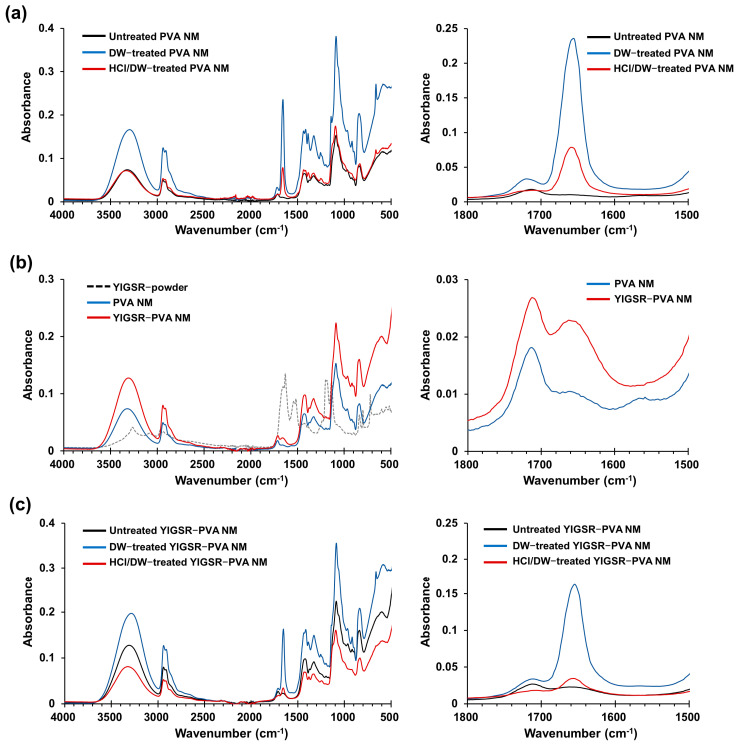
FTIR spectra of peptide-blended PVA NMs. Electrospun PVA NM is untreated (DW-treated PVA NM) or treated (HCl/DW-treated PVA NM) with HCl vapor for 2 min, followed by soaking in water for 18 h. The NMs are washed three times with PBS, dried, and then analyzed by FTIR spectroscopy. (**a**) FTIR spectra of HCl-treated PVA NM, (**b**) YIGSR-blended PVA NM, and (**c**) HCl and/or DW-treated YIGSR-PVA NMs.

FTIR spectra of the electrospun PVA, PVA/PAA, and PVA/PAA/GA nanofibers showed characteristic bands in the wavelength range of 3600–3200 cm^−1^ (O-H stretch), 2900–2800 cm^−1^ (C-H stretch), 1750–1700 cm^−1^, and 1100–1000 cm^−1^, respectively (Figure S3). The peaks at a 1750 cm^−1^ wavelength (shown as line a) and 1100–1000 cm^−1^ wavelength (shown as line b) in the PVA/PAA/GA nanofibers were higher and broader than those of the PVA and PVA/PAA nanofibers. The band at 1150 cm^−1^ was suggested to be C-O-C formed through the crosslinking between OH of a typical PVA structure and -C- of GA molecules; the band at 1720 cm^−1^ was indicative of a C=O bond, which is characteristic of GA [42] Water-treated PVA NMs showed an increased intensity of peaks in the wavelength range of 3600–3200 cm^−1^, 2900–2800 cm^−1^, 1750–1700 cm^−1^, and 1100–1000 cm^−1^, owing to hydrophilic interactions between the hydroxyl groups in PVA and H_2_O (Figure 2a). In comparison, pretreatment of the PVA NM with HCl vapor significantly blocked the increase in these peaks caused by water treatment. Exposure of the electrospun PVA nanofibers to HCl vapor alone, without water treatment, also decreased these peaks compared to that of the untreated control PVA NM (Figure S4). The spectra of the YIGSR-PVA NM showed no significant shifts in the frequency of the characteristic functional groups, except for a slightly higher band at 1650 cm^−1^ when compared with the control PVA nanofiber (Figure 2b). The water treatment of the YIGSR-PVA NM showed changes similar to those observed in the water-treated PVA NM, and the hydrophilic bond between PVA and H_2_O resulted in overlapped bands at 1650 cm^−1^ (Figure 2c). We observed similar FTIR findings for the water-stable RGD-PVA and IKVAV-PVA NMs compared to that for the control PVA NM. Thus, the bands formed by chemical crosslinking in the PVA NM can hardly be detected on hydrophilic bonding with H_2_O, and the increase in the hydrophilic interaction can be blocked by exposing the nanofibers to HCl vapor, resulting in the maintenance of the nanofiber structure in water.

### 3.5. Cell Culture on PVA NMs

**Figure 3:**
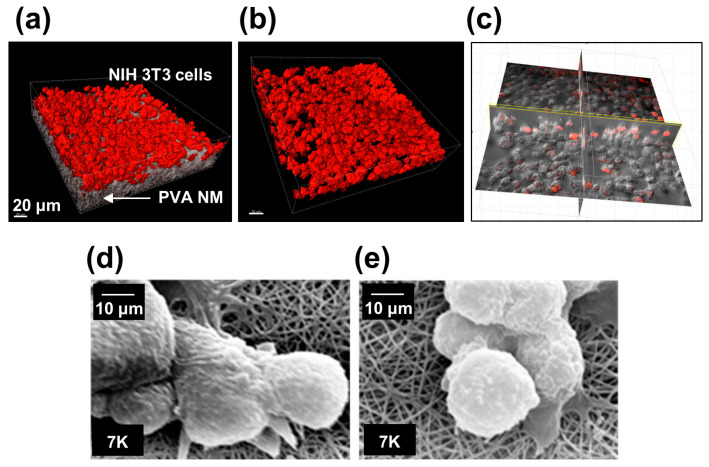
The 3D adhesion of cultured cells on PVA NMs. (**a**) CellTracker Red-labeled NIH 3T3 cells on PVA NM. (**b**) Cells alone without PVA NM imaging. (**c**) Cross-sectioned view of cells on PVA NM analyzed using a confocal microscope. Images are shown using the surface function of Imaris software. (**d**,**e**) NIH 3T3 and MLE-12 cells are cultured on HCl vapor-treated PVA NM and observed using SEM.

When NIH 3T3 cells were cultured on water-soluble PVA, PVA/PAA, and PVA/PAA/GA NMs that were not exposed to HCl vapor, most cells tended to aggregate on the membrane 12 h after culture and did not adhere to the membrane. Unbound cells formed aggregates on the membrane 24 h after culture, and most of the cells floated 48 h after culture. We tested whether cells seeded on the water-stable PVA NM adhered to and grew three-dimensionally on the membrane. NIH 3T3 cells were labeled with CellTracker Red and cultured for 12 h on the PVA NM. 3D imaging analysis showed an even distribution of fluorescence-labeled NIH 3T3 cells adhering to the PVA NM (Figure 3a and Video S1). The cultured cells on the PVA NM showed a round shape rather than the flattened shape observed in the culture of cells on a culture plate (Figure 3b). Cross-sectional image analysis shows the infiltration of cultured cells in the PVA NM (Figure 3c and Video S2). The SEM images of cultured NIH 3T3 and MLE-12 cells on the PVA NM show the attachment of cells to well-preserved PVA nanofibers in the culture medium and aggregation of several cells surrounding the attached cells to the surface of the membrane (Figure 3d,e). A close view of the cell–nanofiber interactions shows that the projected areas of cells attached to the nanofibers and the cultured cells on the PVA NM grew three-dimensionally.

### 3.6. Cell Culture on Peptide-Blended PVA NMs

**Figure 4:**
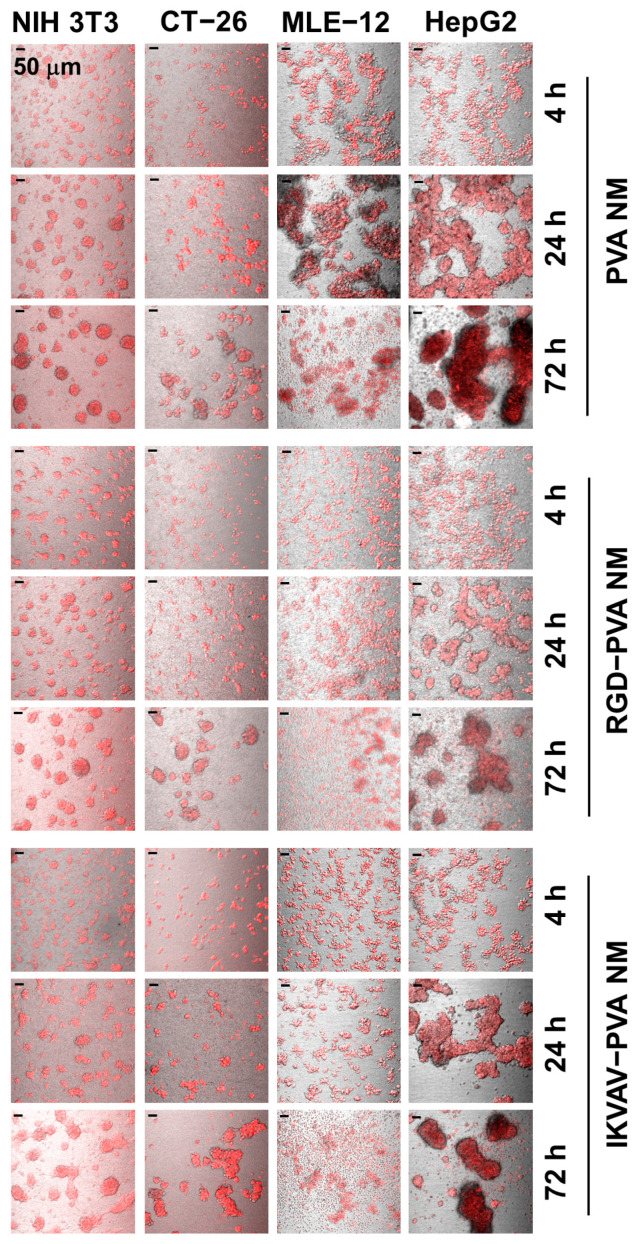
Adhesion of cultured cells on PVA NMs. Merged fluorescence and DIC images of cells labeled with CellTracker Red and cultured for the indicated times on the membranes.

The transparency of the PVA NM facilitated the observation of cultured cells in the DIC image as on a culture plate. Several types of cells, including NIH 3T3, CT-26, MLE-12, and HepG2 cells, were labeled with CellTracker Red and seeded on the PVA NM. Merged images of fluorescence and DIC show that 80% of seeded cells adhered to the membranes 4 h after seeding; however, cell aggregates on the membranes increased after prolonged culture (Figure 4). Among the tested cells, MLE-12 lung epithelial cells and HepG2 hepatoma cells on the PVA NM easily formed cell aggregates owing to the lower adhesion of epithelial-like cells to the membrane than NIH 3T3 and CT-26 cells at all culture times. The physical characteristics of PVA nanofibers facilitate the adherence of cells to the membrane, but their binding to nanofibers is weak. Thus, cells on the PVA NM may form aggregates as the culture time increases. Live imaging of MLE-12 cells cultured on the PVA NM shows that the cultured cells tended to grow in clusters (Video S3). In comparison, the cells on the culture plate adhered to and extended along the surface (Video S4). Next, we investigated whether the peptide-blended PVA NM could provide better attachment of cells to the nanofibers, similar to an ECM in the tissue microenvironment. The peptide-blended PVA NM was produced by electrospinning a PVA solution containing peptides, such as RGD and IKVAV. Unexpectedly, the aggregation of cultured NIH 3T3, CT-26, and HepG2 cells on the RGD-PVA and IKVAV-PVA NMs increased with time. This result indicates that the attachment of cultured cells to the peptide-blended PVA NMs did not significantly increase compared to that on the PVA NM. In comparison, we observed fewer MLE-12 cells without forming large aggregates on the peptide-blended PVA NM than on the PVA NM because of cell death after less adherence and floating of the cultured cells after 24 h of culture. Thus, the blended peptides failed to enhance cell adherence to the nanofibers because the peptides may have been released from the nanofibers into the media or been unexposed to the nanofibers, leading to their inability to bind to the cultured cells.

### 3.7. Treatment of Peptide-Blended PVA NMs with NaOH

**Figure 5:**
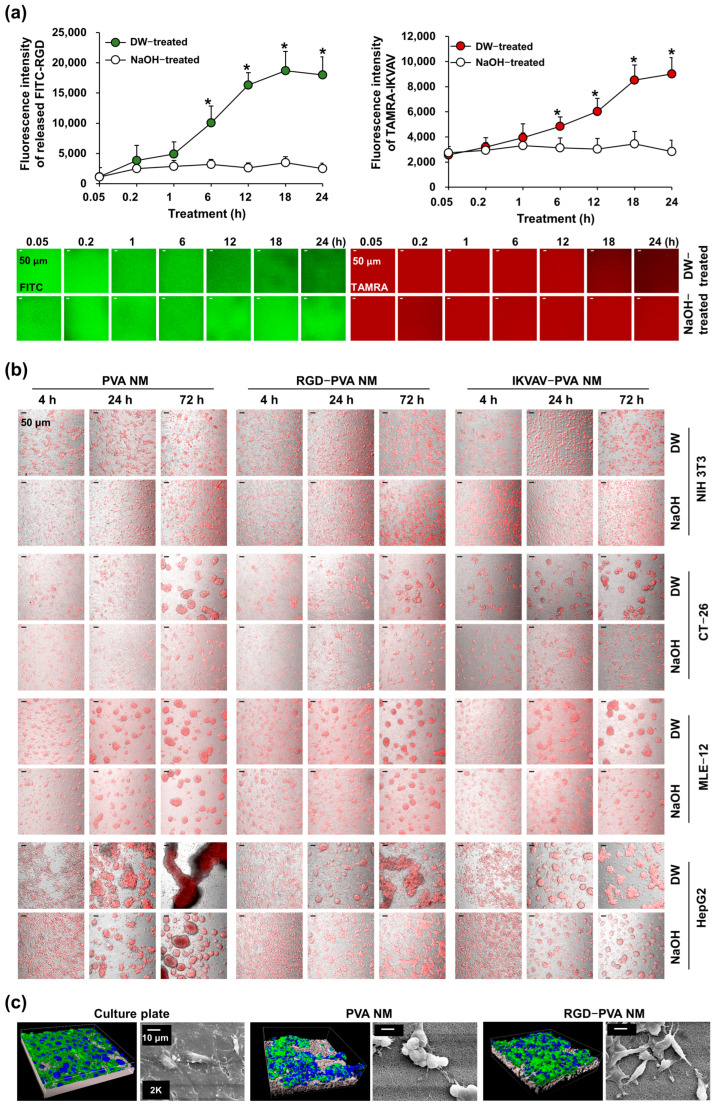
Effects of NaOH treatment on peptide release and cell adhesion in peptide-blended PVA NMs. (**a**) Fluorescence levels in the media measured by fluorescence spectrometry and the membranes measured by confocal microscope. (**b**) Merged fluorescence and DIC images of fluorescence-labeled cells. (**c**) Images shown using the surface function of Imaris software and SEM. * *p* < 0.05 versus 0.05 h treatment.

We evaluated whether peptides were retained in the peptide-blended PVA NM by measuring the fluorescence intensity in the solution and membrane after soaking the fluorescence-conjugated peptide-blended PVA NM in distilled water. As shown in Figure 5a, the fluorescence in the solution significantly and gradually increased up to 24 h after treating the FITC-conjugated RGD-PVA and TAMRA-conjugated IKVAV-PVA NMs with distilled water. In addition, confocal microscopy reveals decreased fluorescence in both PVA NMs after water treatment. Next, we tested whether the alkali or acidic treatment of PVA NMs affected the release of blended compounds in the nanofibers because crosslinked PVA may be stabilized in highly acidic and alkaline environments [31]. When the fluorescence-conjugated peptide-blended PVA NM was treated with 1 M NaOH, fluorescence was not released into the solution, and the uniformly detected fluorescence in the PVA NM did not decrease 24 h after treatment. In comparison, the treatment of NMs with 1 M HCl failed to block the release of fluorescence.

### 3.8. Cell Culture on NaOH-Treated PVA NMs Blended with Peptides

When the FITC-conjugated RGD-PVA NMs were treated with NaOH for 12 h, dried, and soaked in water for 24 h, the fluorescence did not significantly increase in the solution after the second soaking of the membranes. The peptide-blended PVA NMs were treated with distilled water and 1 M NaOH for 12 h, washed with culture media, and used to culture CellTracker Red-labeled NIH 3T3, CT-26, MLE-12, and HepG2 cells. We observed a significant increase in the size of cell aggregates in the distilled water-pretreated RGD-PVA and IKVAV-PVA NMs but not in the NaOH-pretreated membranes (Figure 5b). The 3D analysis of confocal microscopic images and SEM show the layered adhesion of cultured NIH 3T3 cells to NaOH-treated RGD-PVA and IKVAV-PVA NMs. In contrast, the cells grown on the NaOH-treated control PVA NM and culture plate tended to grow in clusters and flattened monolayers, respectively (Figure 5c).

### 3.9. Adhesion of Seeded Cells to Peptide-Retained PVA NMs

**Figure 6:**
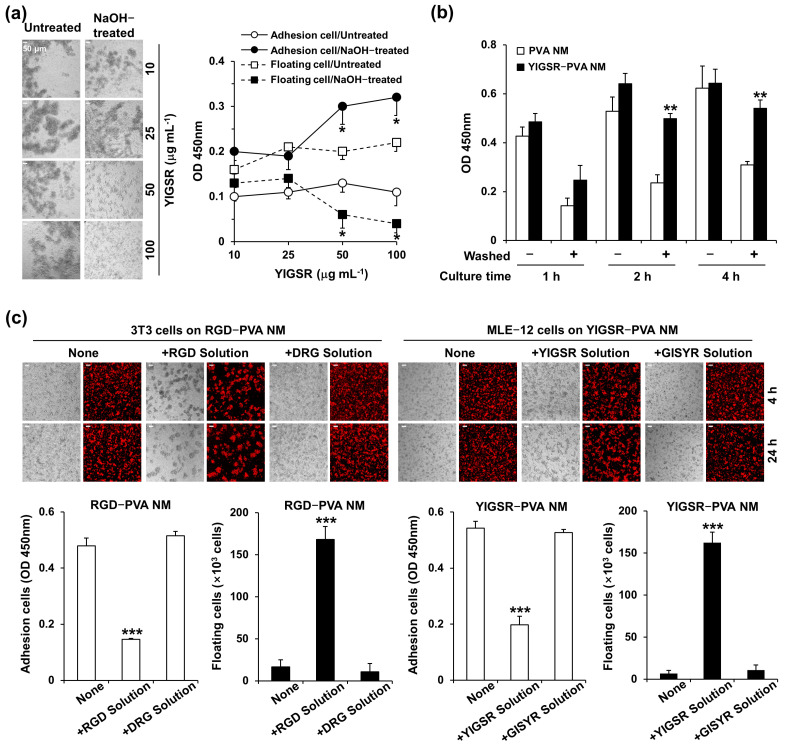
Culture of cells on peptide-retained PVA NMs. (**a**) DIC images of the cells and CCK-8 assay of viable cells in culture media and attached to NMs. (**b**) CCK-8 assay of viable cells adhered to the membranes. (**c**) DIC and fluorescence images using a confocal microscope and CCK-8 assay of viable cells. * *p* < 0.05 versus untreated. ** *p* < 0.05 versus PVA NM. *** *p* < 0.05 versus none.

Among the tested cells, the adhesion of MLE-12 cells to PVA NMs was lower than that of other cell types during the entire culture period. We conducted cell adhesion assays to examine the ability of MLE-12 cells to adhere to peptide-blended PVA NMs. First, YIGSR-PVA NMs blended with various peptide concentrations were produced and treated with 1 M NaOH for 12 h. MLE-12 cells were cultured for 24 h on the untreated and NaOH-treated YIGSR-PVA NMs. Figure 6a shows a gradual decrease in the formation of cell aggregates in NaOH-treated YIGSR-PVA NMs with increasing concentrations of YIGSR but a similar formation of cell aggregations in untreated YIGSR-PVA NMs with high peptide concentrations. In the CCK-8 assay of the cells cultured on the NaOH-treated YIGSR-PVA NMs with a higher concentration of YIGSR, we observed the adherence of more cells to the surface of the membrane and fewer floating cells in the culture media. This result suggests that more MLE-12 cells bind to the YIGSR peptide-retained YIGSR-PVA NMs. Second, MLE-12 cells were incubated for 4 h to enable the adherence of seeded cells to the surface of the NaOH-treated control PVA and YIGSR-PVA NMs, and the plates were placed on an orbital shaker at 50 rpm (washed) and not subjected to shaking (without washing) for 10 min after the indicated times of culture. Cells that adhered to the membranes were collected after removing unbound floating cells from the media by gentle shaking. The number of adhered cells in the YIGSR-PVA NMs significantly increased compared to that in the control PVA NM when the cells were washed after 2 h of incubation (Figure 6b). The total number of cells obtained without washing was similar for the PVA and YIGSR-PVA NMs. Finally, we conducted a competitive assay using soluble peptides to evaluate whether the peptides retained in the nanofibers mediated the adherence of cells to NaOH-treated RGD-PVA and IKVAV-PVA NMs. Adding soluble RGD and YIGSR (100 μg/mL) peptides to the culture medium decreased the adhered NIH 3T3 and MLE-12 cells to RGD-PVA NMs and YIGSR-PVA NMs, respectively (Figure 6c). In contrast, DRG and Gly-Ile- Ser-Tyr-Arg (GISYR) peptides, scrambled peptides of RGD and YIGSR, failed to block the adherence of the cells to peptide-blended PVA NMs. We also observed a similar blocking effect of the IKVAV peptide but not the VIAKV peptide on the adherence of MLE-12 cells to IKVAV-PVA NMs). These results indicate that the free form of the peptides prevented the interaction of cells in the peptide-retained nanofibers. We tested whether NaOH treatment affected the covalent bonds between the peptides and the crosslinked PVA molecules. In the FTIR spectra of the PVA and YIGSR-PVA NMs, NaOH treatment increased absorbance at 1600–1550 cm^−1^, corresponding to the C=C bond, but decreased absorbance at 1750–1700 cm^−1^, the peak for the C=O bond, compared to that of membranes after water treatment (Figure S5).

**Figure 7:**
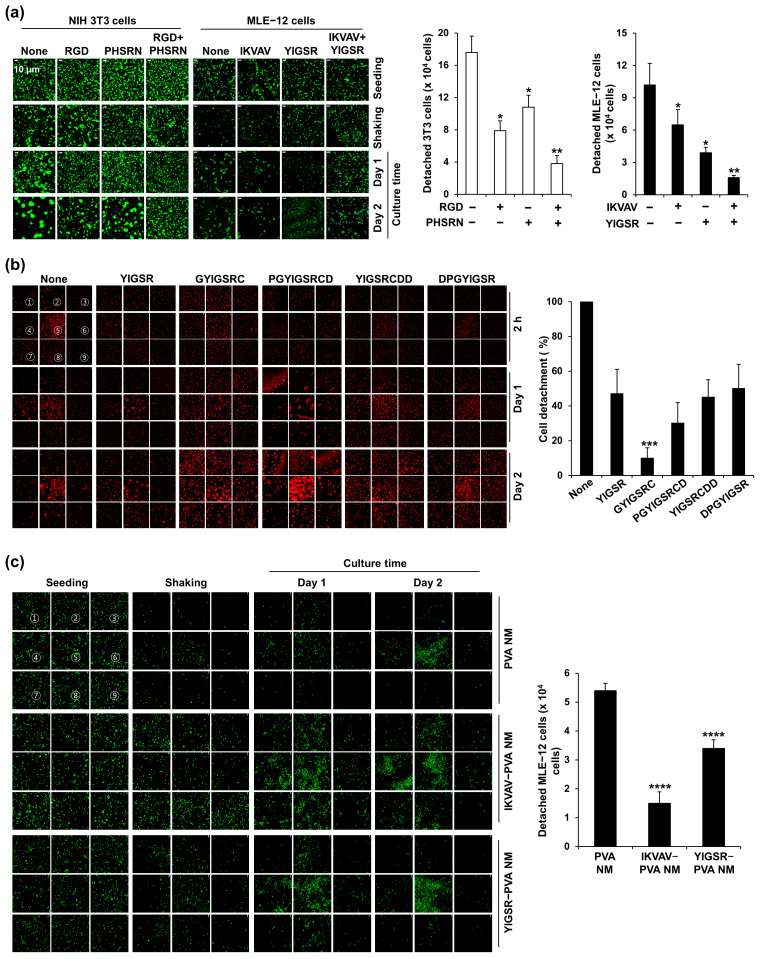
Culture of cells on PVA NMs containing various types of peptides. (**a**) NIH 3T3 and MLE-12 cells, (**b**) MLE-12 cells, and (**c**) primary colon epithelial cells observed using a confocal microscope and numbers of detached cells were counted based on 9 different areas (marked from 1 to 9). * *p* < 0.05 versus without peptides. ** *p* < 0.05 versus with one type of peptide. *** *p* < 0.05 versus YIGSR. **** *p* < 0.05 versus PVA NMs.

Next, we produced PVA NMs containing two different peptides: RGD+PHSRN and IKVAV+YIGSR. NIH 3T3 and MLE-12 cells were seeded on RGD+PHSRN-PVA NMs and IKVAV+YIGSR-PVA NMs, respectively, washed with gentle shaking, and cultured for two days. As shown in Figure 7a, the two different peptide-blended PVA NMs further increased the adhesion of NIH 3T3 and MLE-12 cells compared to that of the peptide-blended PVA NMs. Moreover, we produced various PVA NMs blended with different forms of peptides containing the YIGSR sequence found in laminin protein, such as Gly-Tyr-Ile-Gly-Ser-Arg-Cys (GYIGSRC), Pro-Gly-Tyr-Ile-Gly-Ser-Arg-Cys-Asp (PGYIGSRCD), Tyr-Ile-Gly-Ser-Arg-Cys-Asp-Asp (YIGSRCDD), and Asp-Pro-Gly-Tyr-Ile-Gly-Ser-Arg (DPGYIGSR). To rule out the detection of a higher aggregation of cultured MLE-12 cells in one area of the PVA NM 24 h after culture, we examined the adherence of CellTracker Red-labeled cells to nine different membrane areas (Figure 7b). In addition, the number of floating cells harvested from the culture media was counted. Among the various PVA NMs tested, cell adherence was the most potent in the GYIGSRC-PVA NM. We tested whether the adherence of primary epithelial cells increased in peptide-blended PVA NM compared to that in the control PVA NM. Fewer primary colon cells adhered to the PVA NM and formed more aggregates on the PVA NM than on the culture plate (Figure S6). In comparison, when primary colon cells were seeded on IKVAV-PVA and YIGSR-PVA NMs washed with gentle shaking and cultured for two days, more cells adhered to the peptide-blended PVA NMs than to the control PVA NM (Figure 7c). Taken together, the peptide retained in the nanofibers after NaOH treatment increased the adhesion of cells to the nanofibers.

### 3.10. Comparison of Cell Growth on Peptide-Blended PVA NM and ECM-Coated PVA NM

**Figure 8:**
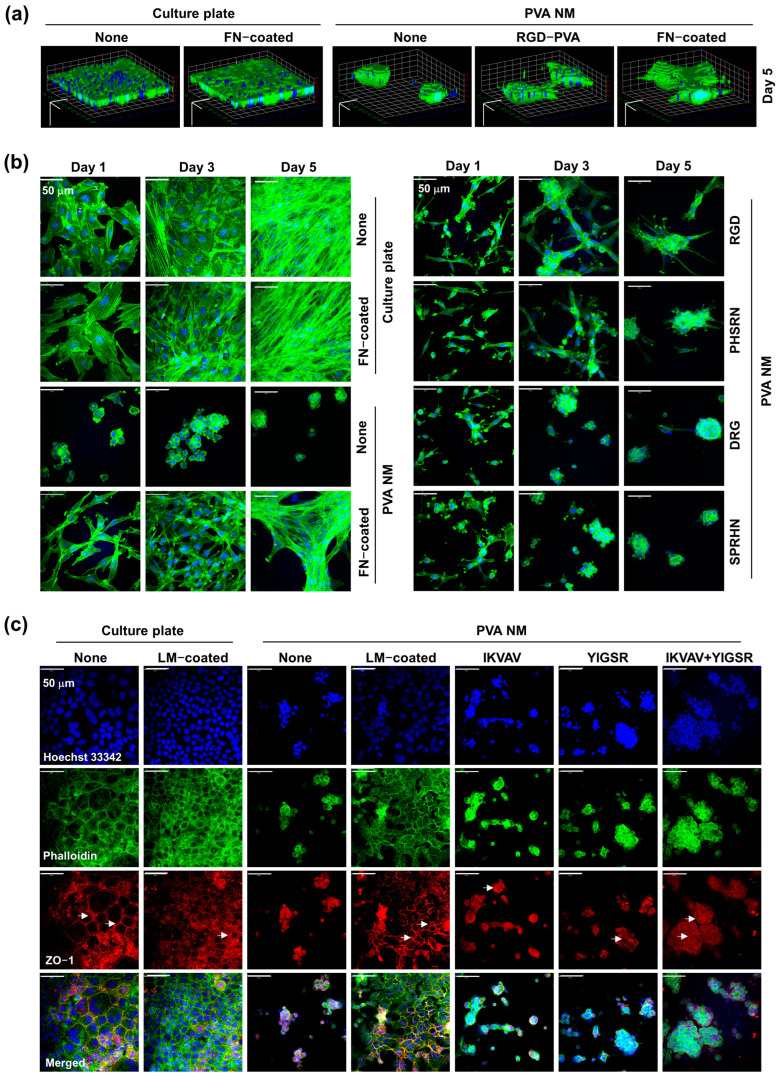
Pattern of cell growth on ECM protein-coated and peptide-retained PVA NMs. (**a**) Imaris view of confocal microscopic images. The scale bar is 50 μm. (**b**) NIH 3T3 and (**c**) MLE-12 cells shown in confocal microscopic images, with cell nuclei (Hoechst 33342, blue), actin microfilaments (Alexa Flour 488 Phalloidin, green), zona occludin-1 (ZO-1, red) Arrows indicate ZO-1 expression.

To provide in vivo-like ECM conditions for cell growth in vitro, we produced the peptide-blended PVA NM rather than the protein-blended PVA NM because of the denaturation of proteins by the chemical crosslinking agent added during the nanofiber manufacturing process. Thus, we compared the cell growth on the peptide-blended PVA NM with that on the culture plate and the PVA NM coated with cell-binding ECM proteins. As shown in Figure S7, soluble fibronectin coated on the PVA NM was detected along the PVA nanofiber but not as a gel on the PVA NM after fluorescence staining of the proteins. The DIC imaging of NIH 3T3 cells obtained after five days of culture show that cell growth on the fibronectin-coated PVA NM was similar to that on uncoated and fibronectin-coated culture plates (Figure S8). NIH 3T3 cells cultured on the RGD-PVA NM and fibronectin-coated PVA NM displayed significantly fewer cell clusters than those on the control PVA NM. In addition, MLE-12 cells cultured on the IKVAV-PVA NM displayed a distribution of cells similar to those on the laminin-coated PVA NM compared to those on the control PVA NM. In a 3D analysis of the confocal microscopy images, the growth pattern of NIH 3T3 cells on the RGD-PVA NM showed a trend in between that on the PVA NM and the fibronectin-coated PVA NM (Figure 8a). Next, we examined the cytoskeletal organization of NIH 3T3 cells cultured on the three different PVA NMs by actin staining. Actin stress fiber formation in cells on the fibronectin-coated PVA NM significantly increased with time, similar to that in the uncoated control and fibronectin-coated culture plates (Figure 8b). NIH 3T3 cells cultured on RGD-PVA and PHSRN-PVA NMs showed a projectile distribution of actin filaments resembling cells cultured on the fibronectin-coated PVA NM during cell spreading. In contrast, the cells on the control PVA NM and scrambled peptide-blended PVA NMs formed round aggregates. As shown in Figure 8c, MLE-12 cells cultured on the laminin-coated PVA NM exhibited a distinct spreading cell morphology, similar to the cells cultured on the laminin-coated culture plate. Zonula occludens (ZO)-1, a tight junction protein, was observed on the contact surfaces between the cultured cells on the laminin-coated PVA NM and the culture plate (arrows indicate). The spreading of MLE-12 cells increased in IKVAV-PVA and YIGSR-PVA NMs compared to that in the control PVA NM. Cell-to-cell adhesion and expression of ZO-1, but not of cell aggregates, were more evident in the two peptide-blended PVA NMs. These results suggest that peptides in the PVA NM promote cell growth, as detected under ECM-mimicking conditions.

**Figure 9:**
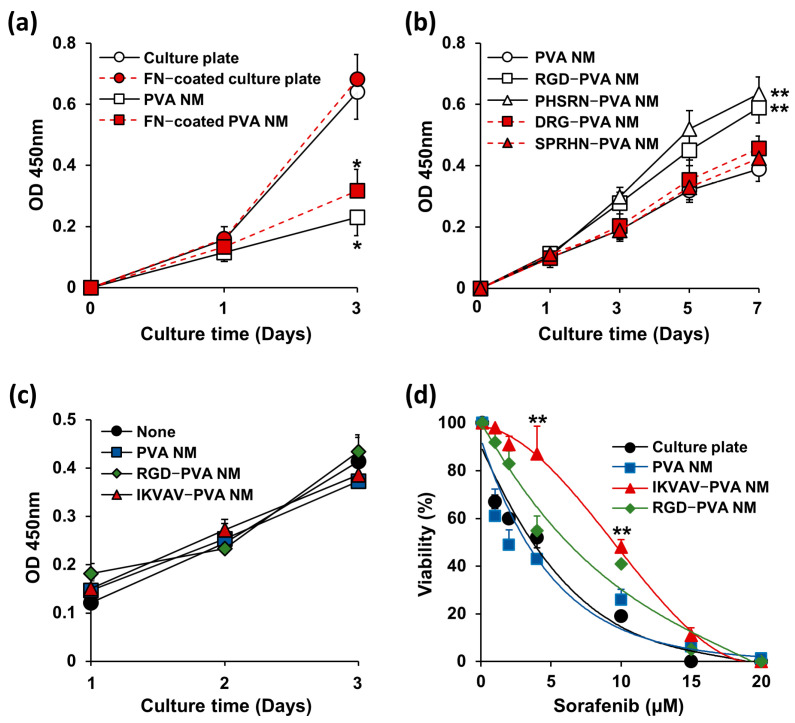
Growth rate of the cells cultured on the peptide-retained PVA NMs. (**a,b**) CCK-8 assay of NIH 3T3 cells. (**c**) NIH 3T3 cells cultured in the conditioned media on the culture plate. (**d**) CCK-8 assay of HepG2 cells. * *p* < 0.05 versus culture plate, ** *p* < 0.05 versus PVA NM.

Next, in parallel with the cell growth pattern, we compared the proliferation rates of the cultured cells on a culture plate, PVA NM, and peptide-blended PVA NM. The viability of cells that adhered to the membranes was measured using a CCK-8 assay. As shown in Figure 9a, the proliferation of NIH 3T3 cells cultured on the PVA NM increased with time; however, fewer cells were detected on the PVA NM than on the culture plate. Fibronectin coating on the culture plate and the PVA NM did not affect the proliferation of NIH 3T3 cells. The growth rate of NIH 3T3 cells on the peptide-blended PVA NMs was similar to that on the control PVA NMs (Figure 9b). We tested whether the lower growth rate of cells on the PVA NM than on a culture plate was due to the toxicity of the PVA nanofibers. Conditioned media were obtained after soaking the pieces of the tested PVA NM (1.3 × 1.1 cm^2^) in the culture media (700 μL) for seven days. When NIH 3T3 cells were cultured for three days on a culture plate in conditioned media obtained after soaking the membranes, there was no significant inhibitory effect on the cell viability (Figure 9c). We further evaluated the cytotoxic response of cancer cells cultured in a culture plate and PVA NMs with a chemotherapeutic agent. When HepG2 cells were treated with an increasing dose of sorafenib on a culture plate, PVA NM, IKVAV-PVA NM, and RGD-PVA NM, the IC_50_ values were 3.94 ± 0.48, 3.71 ± 0.12, 9.05 ± 0.63, and 6.62 ± 0.42 µM, respectively (Figure 9d). HepG2 cells cultured on the IKVAV-PVA NM demonstrated greater resistance than those cultured on a culture plate and the PVA NM. These results suggest that PVA and peptide-blended PVA nanofibers do not secret cytotoxic compounds, and the lower cell growth rate on the PVA NM may be due to the slow growth of cells under 3D culture conditions.

### 3.11. Bioinert of Peptide-Blended PVA NM

**Figure 10:**
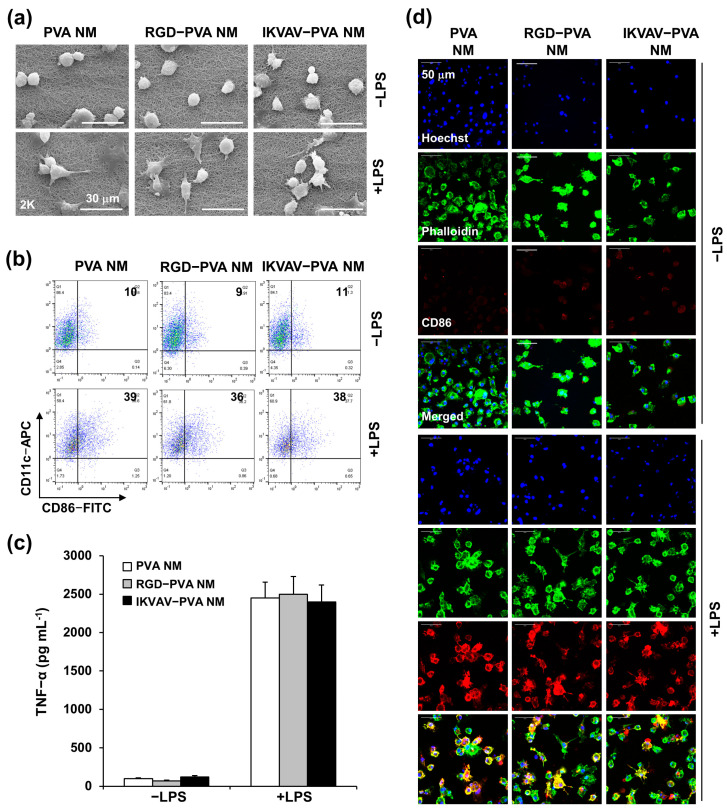
Culture of BMDCs on peptide-blended PVA NMs. (**a**) Cell morphology assessed using SEM. (**b**) Expression level of CD86 measured by flow cytometry. (**c**) The concentration of TNF-α measured by ELISA. (**d**) Cell morphology and CD86 expression evaluated by confocal microscopy (cell nuclei (Hoechst 33342, blue), actin microfilaments (Alexa Flour 488 Phalloidin, green), CD86 (red)).

PVA is a biocompatible polymer [28]. We tested whether culturing immune cells, such as DCs, on PVA and peptide-blended PVA NMs affected their activation status. The adhesion, morphology, and activation of BMDCs were evaluated 24 h after seeding the cells on a culture plate and NaOH-treated PVA NMs. The SEM micrographs show that unstimulated BMDCs maintained a round shape on the culture plates, whereas LPS-stimulated BMDCs were spread on the surface of the plate (Figure S9a). In comparison, RGD and IKVAV did not induce changes in the BMDC morphology. Inactive DCs expressed low levels of DC activation markers, such as CD86, on the cell surface, whereas activated DCs expressed high levels of CD86 [37]. Flow cytometry and confocal analyses of BMDCs cultured with the peptides reveal no increase in CD86 levels (Figure S9b,c). Similarly, BMDCs did not produce tumor necrosis factor (TNF)-α in the presence of the peptides (Figure S9d). Therefore, these peptides did not activate DC. Next, when BMDCs were cultured on PVA NMs, the SEM showed attachment of the cells to the surface of all tested PVA NMs (Figure 10a). Unstimulated DCs showed no cytoplasmic projection on PVA and peptide-retained PVA NMs, whereas LPS-stimulated BMDCs on all membranes showed more pronounced cytoplasmic projections than unstimulated cells. Similarly, BMDCs cultured on PVA, RGD-PVA, and IKVAV-PVA NMs showed low percentages of CD86-positive cells among total cultured cells (12 ± 3%, 14 ± 5%, and 12 ± 3%, respectively) compared to cells on a culture plate (15 ± 4%) (Figure 10b). CD86 expression levels in LPS-stimulated BMDCs cultured on peptide-blended PVA NMs were similar to those in the control PVA NM. In addition, the secretion of TNF-α was not significantly increased when BMDCs were cultured on RGD-PVA and IKVAV-PVA NMs compared to the control PVA NM (Figure 10c). There was no significant difference in the secretion of TNF-α by LPS-stimulated BMDCs in all conditions. We also obtained similar results in the secretion of other proinflammatory cytokines, such as IL-1β and IL-6 Confocal analysis shows CD86-negative BMDCs on the peptide-retained PVA NM (Figure 10d). LPS stimulation increased the number of CD86-positive BMDCs in all tested PVA NMs. These results suggest that the peptide-retained PVA NMs were non-reactive with DCs.

## 4. Discussion

Water is used as the sole solvent of PVA for electrospinning, but producing PVA nanofibers using water alone is poor. In this study, the nanofiber structure in the post-crosslinked PVA NM remained intact even after treatment with water. More importantly, water stability was preserved by blending various peptides into PVA nanofibers. The crosslinked PVA NM maintained optical transparency in water after repetitive wetting and drying of the membrane. The crosslinking effectiveness and swelling behavior of the PVA nanofibers may depend on several factors. Crosslinking to the hydroxyl groups in PVA rendered the nanofibers water-insoluble [31,32,43]. To find the optimum condition for preparing non-dissolved and optically transparent PVA nanofibers in solution, we fabricated different PVA nanofibers: PVA/PAA and PVA/PAA/GA. The addition of PAA to PVA increases the stability and crystalline structure of the nanofibers via chemical crosslinking [30,44]. PAA is used to overcome the instability of water-soluble PVA because the carboxyl groups in PAA can react with the hydroxyl groups in PVA to form covalent ester bonds [30,44,45]. Second, a crosslinking reaction occurs between the hydroxyl group in PVA and the aldehyde group in GA [31]. However, the fabrication of PVA and GA via electrospinning is complex and poor because of the rapidly increasing viscosity of the solution [32]. Thus, post-treatment of the PVA nanofibers with GA was performed by immersing the PVA nanofiber mat in a GA solution or GA vapor [43,46]. The GA vapor-treated PVA nanofiber mat was water-insoluble but generated excess unreacted aldehyde functional groups [46]. Gough et al. demonstrated that GA components on the surface of a nanofiber mat induced apoptosis of cultured cells on a collagen–PVA film crosslinked by GA [47]. In this study, PVA nanofibers crosslinked by GA alone lost their morphology in water. However, the addition of GA to PVA/PAA increased the water stability of the post-crosslinked nanofibers, and the crosslinked PVA/PAA/GA nanofibers were not cytotoxic. PAA may act as an acidic catalyst for the crosslinking reaction between PVA and GA. Destaye et al. reported that the crosslinking reaction of PAA and GA was due to the intermolecular and intramolecular acetal bridges between the hydroxyl groups in PVA/PAA and the aldehyde molecules of GA [46]. Therefore, simple chemical crosslinking of PVA with PAA and GA provided more water-stable PVA nanofibers. Third, after fabrication with PVA/PAA/GA, the fabricated membrane was post-crosslinked by exposure to HCl vapor. The integrity of the post-crosslinked PVA/PAA/GA nanofibers was maintained for a prolonged period after repetitive treatment, with no fiber deformation in the solution. The FTIR results of the HCl vapor-treated PVA NM show the loss of peaks observed after hydrophilic interaction between the hydroxyl groups in hydrophilic PVA and H_2_O. The formation of insoluble PVA nanofibers by HCl vapor may be due to several physical or chemical changes, including deacetylation, dehydration, and increased hydrogen bonding in the PVA [39]. We speculate that the HCl treatment resulted in increased crosslinking with the loss of the unreacted alcohol groups of PVA in the crosslinked PVA/PAA/GA nanofibers, possibly due to hydrogen bonding.

ECM proteins provide locations for cell adhesion, and binding surface receptors accomplish cell–substrate adherence. In addition, ECM fibers, such as collagen, control cell migration and adhesion through differences in fiber density and diameter [48]. Similarly, the growth of cells cultured on a nanofibrous scaffold may depend on the physical properties of the nanofiber, including fiber diameter, porosity, and pore size distribution [49,50,51]. The mean diameter of the PVA nanofibers was around 180–220 nm, which is similar to that of collagen fibrils with dry diameters between ~100 nm and ~300 nm [52]. Thus, the adherence of seeded cells to the fibers on the PVA NM but not to the solubilized PVA membranes may be due to the physical interaction between the cells and the nanofibers. Deep cellular infiltration into the PVA NM is limited because of the constant fiber diameter and small pore size compared to other nanofibers, such as the polycaprolactone nanofiber scaffold [37]. All tested cells maintain 3D adherence, growth, and survival on the PVA NM. However, cell adhesion was low and non-biological, owing to the lack of specific biological interactions between the cells and nanofibers in the PVA NM. Therefore, the cultured cells formed cell aggregates during prolonged culture, and the aggregation of epithelial cells tended to be higher than that of fibroblasts on the PVA NM. These results suggest that the PVA NM is a suitable scaffold in 3D cell culture but should be modified to be ECM-like in cell culture.

The primary class of surface receptors in the interaction of cells with the ECM is integrins, a family of heterodimeric proteins that bind to a range of ECM constituents, including fibronectin, vitronectin, laminin, and collagen [53,54]. For example, fibronectin and laminin contain specific interaction sites with different integrin receptors, such as RGD, YIGSR, and IKVAV sequences [54,55]. Fibroblasts express fibronectin and laminin receptors [54]. The extremely hydrophilic nature of PVA has been reported to prevent cells from adhering to hydrogels; however, they can adhere to a fibronectin-modified PVA hydrogel by the covalent linking of fibronectin to the PVA surface [56]. Another group showed that a PVA–methacrylate hydrogel mixed with Cys-Arg-Gly-Asp (CRGD) peptide increased cell adhesion compared to the gelatin-containing hydrogel [57]. In this study, peptides containing the desired recognition sequences in fibronectin and laminin to bind the integrins of cultured cells were blended in PVA nanofibers to mimic the ability of the ECM to bind cells.

Unexpectedly, the adherence of cultured cells to peptide-blended PVA NMs was not more potent than the control PVA NM. However, after NaOH treatment, many of the incorporated fluorescence-labeled peptides remained trapped in the peptide-blended PVA NM. NaOH treatment enhances cell adhesion and growth in cells cultured on peptide-blended PVA NMs. Moreover, the capability of cells to bind to peptide-blended PVA NMs can be modulated by combining different peptides and peptide sequence lengths to interact with integrins. In this study, peptides were mixed with PVA and crosslinking chemicals without a linker for interactions with PVA. The mechanism of action of NaOH in retaining the blended peptides in crosslinked PVA is yet to be determined. NaOH treatment was used to improve cell adhesion by increasing the hydrophilicity of the nanofibers [58,59,60]. Park et al. reported that NaOH-treated PLGA has larger pores than untreated PLGA [61]. In comparison, NaOH treatment of the PVA nanofibers did not affect the diameter or pore size of the nanofibers. It has been suggested that surface hydrolysis by NaOH results in the presence of oxygen-containing functional groups, including carboxylic and hydroxylic groups, on the polymer surface [62,63]. Therefore, we need further research to reveal the possibility that the increased hydrophilicity caused by NaOH treatment of crosslinked PVA nanofibers containing water-soluble peptides results in the binding of peptides and PVA.

## 5. Conclusions

The creation of an ideal matrix for ECM replacement in a 3D cell culture provides in vivo-like ECM protein–cell interactions. PVA can be used as a polymer-containing bioactive material. However, because of its high hydrophilicity, water-stable PVA nanofibers with functional materials for the development of biomaterials for cell culture and tissue engineering have not been produced. In this study, to improve the water stability of PVA nanofibers, a solution of PVA and PAA was combined with GA and electrospun, followed by post-crosslinking with HCl vapor. The unmodified crosslinked PVA NM supported the attachment of various cell types. Nonetheless, the resulting nanofibers lack cell-binding substrates, rendering them unsuitable for in vivo-mimicking 3D cultures. Cell–ECM interactions are crucial for maintaining cell adhesion, growth, and other functions. Peptides with integrin receptor-binding sequences were blended and retained in the PVA NM, enhancing the 3D binding and growth of cultured cells on the PVA NM. Therefore, electrospun PVA nanofibers blended with peptides still exhibited the physical and biological characteristics of the PVA nanofibers and retained peptides. The peptide-retained PVA NM is non-reactive to cultured DC, indicating that it is bioinert. Using this technique, the PVA NM also includes various kinds of peptides derived from ligands and other molecules to regulate cell functions, such as growth and differentiation in multiple types of cells, as well as cell adhesion. Thus, the peptide-retained PVA NM provides an ECM-like matrix for advancing 3D cell culture, enabling detailed studies on cell behavior, disease mechanisms, and tissue development. A 3D cell culture using a synthetic ECM is a promising way to improve drug development processes in evaluating new drug candidates.

## Data Availability

The original contributions presented in the study are included in the article/Supplementary Material, further inquiries can be directed to the corresponding authors.

## Supplementary Materials

The following supporting information can be downloaded at: https://www.mdpi.com/article/10.3390/jfb15090262/s1, Figure S1: Morphology of peptide-blended PVA NM; Figure S2: Water-contact angles of peptide-blended PVA NM; Figure S3: FTIR spectra of PVA, PVA/PAA, and PVA/PAA/GA nanofibers; Figure S4: FTIR spectra of HCl vapor-treated PVA NM; Figure S5: FTIR spectra of NaOH-treated PVA NM; Figure S6: Culture of primary colon cells on culture plate and PVA NM; Figure S7: Coating of fibronectin on PVA nanofibers; Figure S8: Effects of peptides on the activation of BMDCs; Video S1: A three-dimensional view of CellTracker Red-labeled NIH 3T3 cell cultured on PVA NM, obtained by confocal microscopy; Video S2: Cross-sectional image analyzed using the 3Dl view obtained in Video S1; Video S3: Live images of CellTracker Green-labeled MLE-12 cells cultured in PVA NM; Video S4: Live images of CellTracker Green-labeled MLE-12 cells cultured in an 8-well culture plate.
