# Supplementary material for: Synthetic Extracellular Matrix of Polyvinyl Alcohol Nanofibers for Three-Dimensional Cell Culture
Source: J Funct Biomater. 2024 Sep 10;15(9):262. doi: 10.3390/jfb15090262 (PMC11433135; doi:10.3390/jfb15090262)
Supplement: Supplementary file 1 [file jfb-15-00262-s001.zip › 2024-09-05-supplemetary information-revised.pdf]

Supplementary information

Artificial extracellular matrix of polyvinyl alcohol nanofibers for three-dimensional cell culture

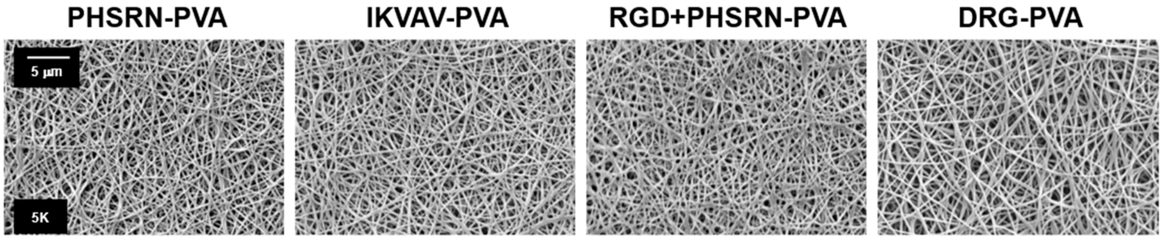

Figure S1 Morphology of **peptide-blended PVA NM.** The structure observed using SEM.

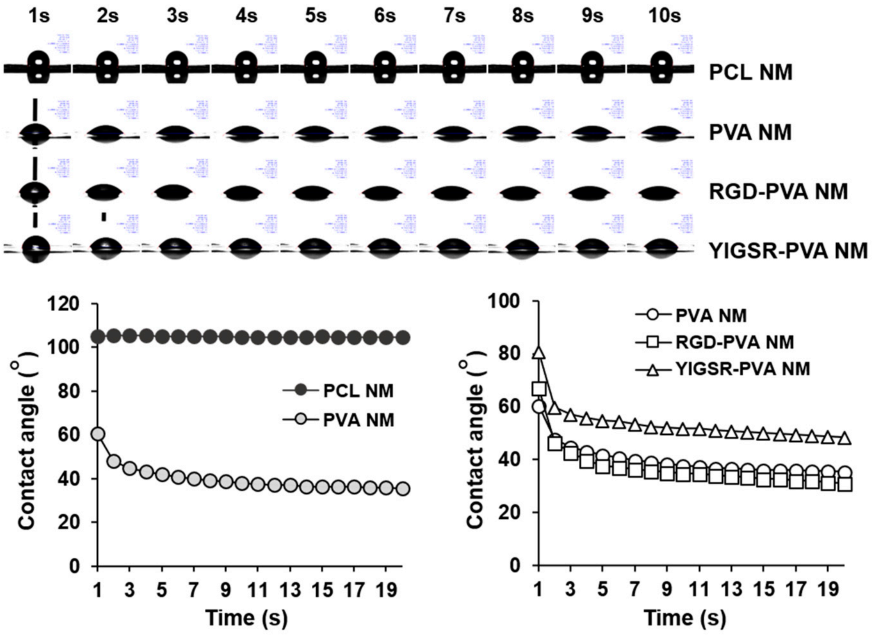

Figure S2 Water-contact angles of **peptide-blended PVA NM.** The contact angles of the electrospun polycaprolactone, PVA, and peptide-blended PVA NMs tested using a drop size analyzer.

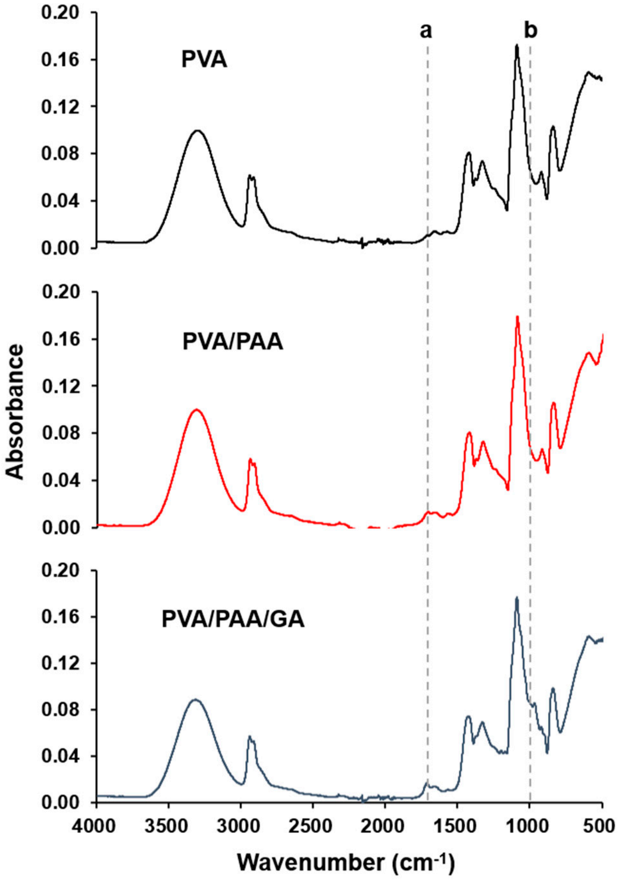

Figure S3 FTIR spectra of PVA, PVA/PAA, and PVA/PAA/GA nanofibers.

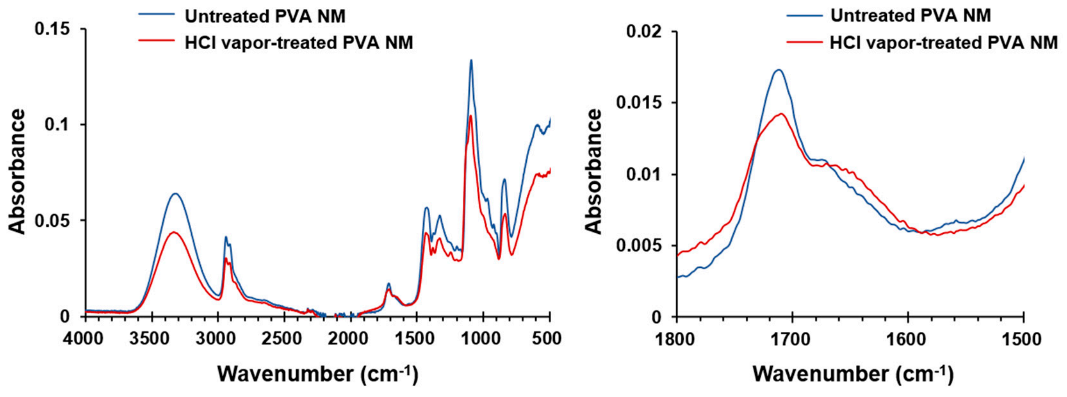

Figure S4 FTIR spectra of HCl vapor-treated PVA NM.

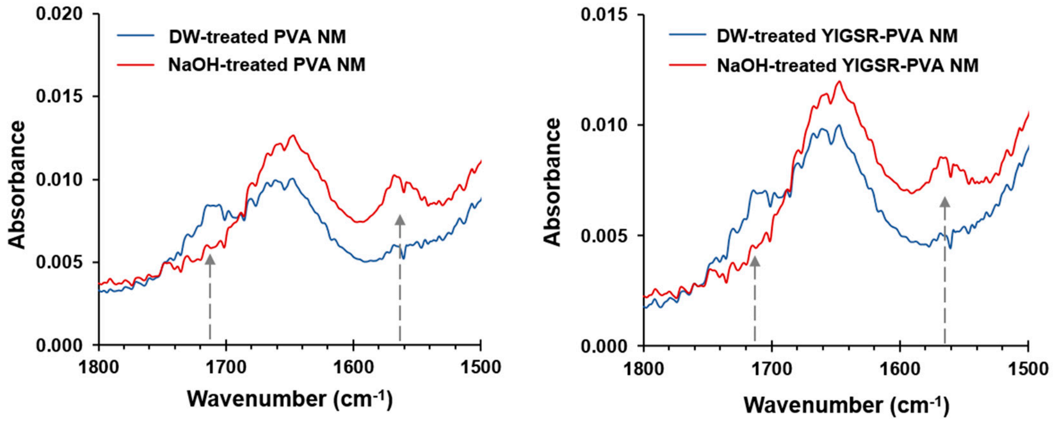

Figure S5 FTIR spectra of NaOH-treated PVA NM.

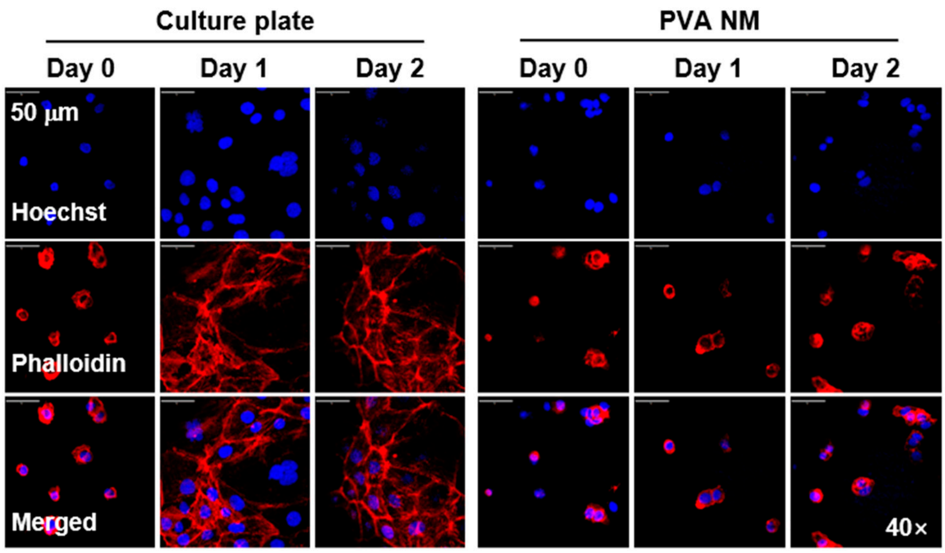

Figure S6 Culture of primary colon cells on culture plate and PVA NM. Confocal microscopic images of the cells stained with Hoechst 33342 and Tetramethylrhodamine isothiocyanate (TRITC)-conjugated phalloidin.

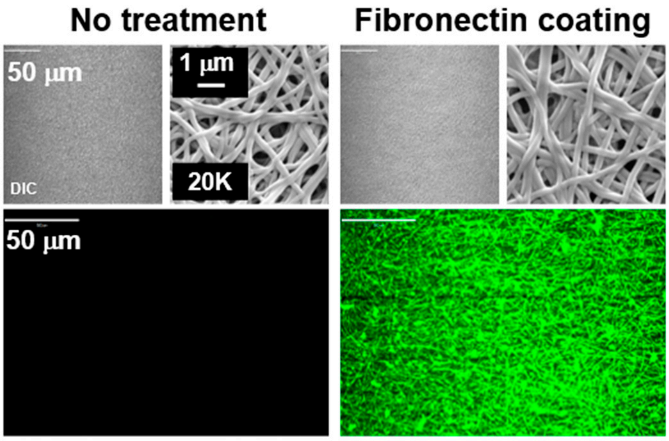

Figure S7 Coating of fibronectin on PVA nanofibers. Fibronectin fluorescently detected after staining with Alexa fluor 488-conjugated anti-rabbit antibody.

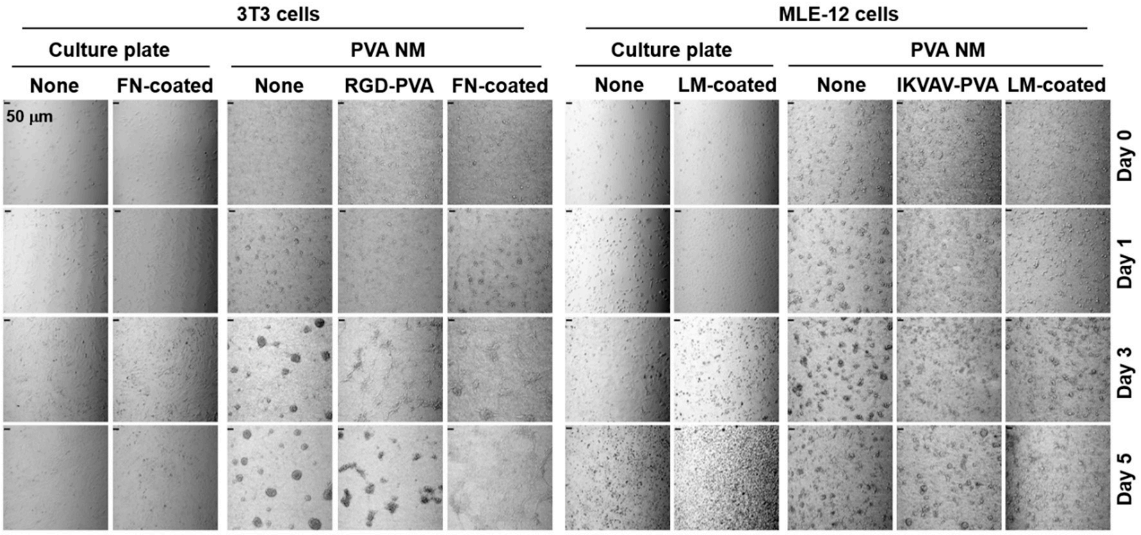

Figure S8 DIC images of cell growth on ECM protein-coated and peptide-retained PVA NMs.

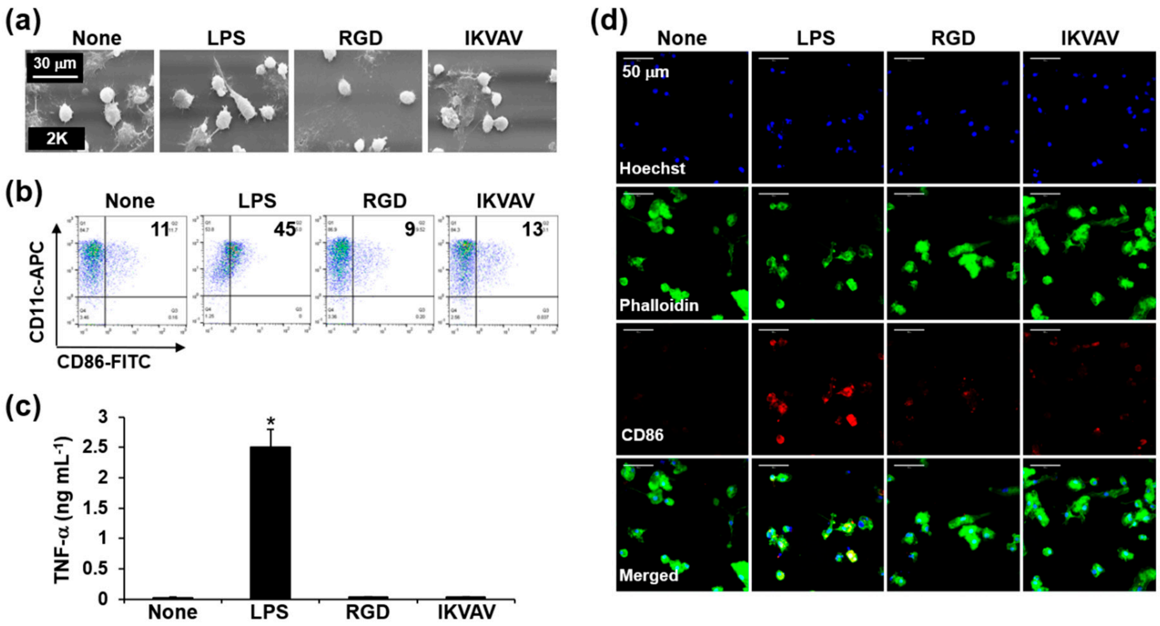

Figure S8 Effects of peptides on the activation of BMDCs. (**a**) SEM, (**b**) flow cytometry, (**c**) ELISA, and (**d**) confocal microscopy after immunofluorescence staining.

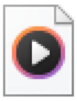

Video S1.mp4

Video S1. A three-dimensional view of CellTracker Red-labeled NIH 3T3 cell cultured on PVA NM, obtained by confocal microscopy.

Video S2.mp4

Video S2. Cross-sectional image analyzed using the 3Dl view obtained in Video S1.

Video S3.mp4

Video S3. Live images of CellTracker Green-labeled MLE-12 cells cultured in PVA NM. Live imaging performed using a ZEISS CellDiscoverer 7 with LSM900 at the 3D Immune System Imaging Core Facility of Ajou University.

Video S4.mp4

Video S4. Live images of CellTracker Green-labeled MLE-12 cells cultured in an 8-well culture plate.
