# Supplementary figures and images for: Synthetic Extracellular Matrix of Polyvinyl Alcohol Nanofibers for Three-Dimensional Cell Culture
Source: J Funct Biomater. 2024 Sep 10;15(9):262. doi: 10.3390/jfb15090262 (PMC11433135; doi:10.3390/jfb15090262)

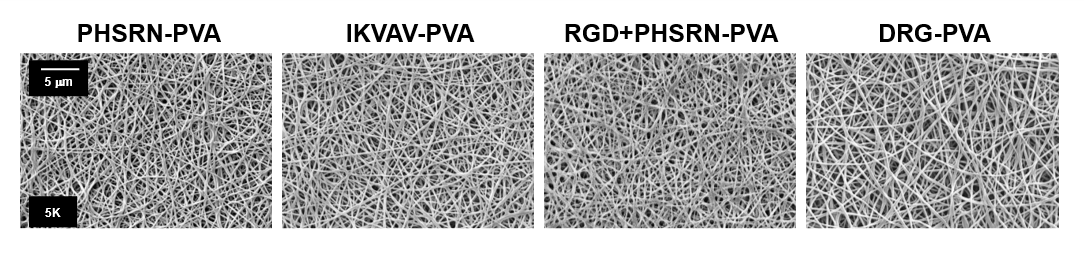

Supplement: Supplementary file 1 [file jfb-15-00262-s001.zip › Fig. S1.png]

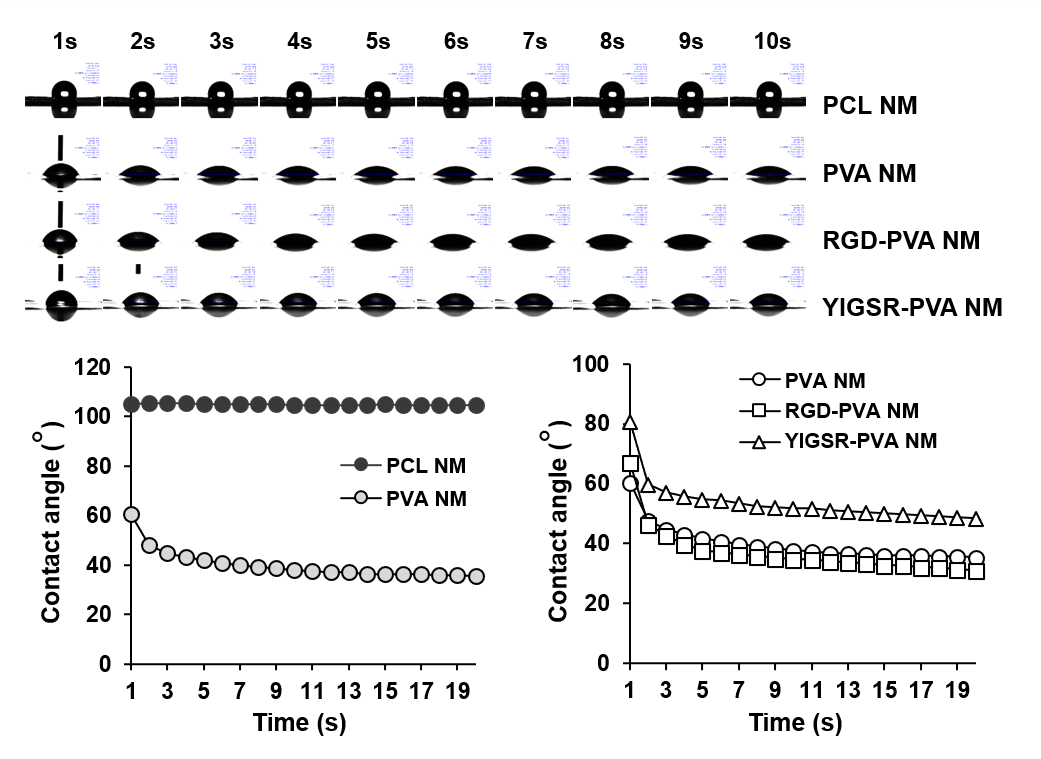

Supplement: Supplementary file 1 [file jfb-15-00262-s001.zip › Fig. S2.png]

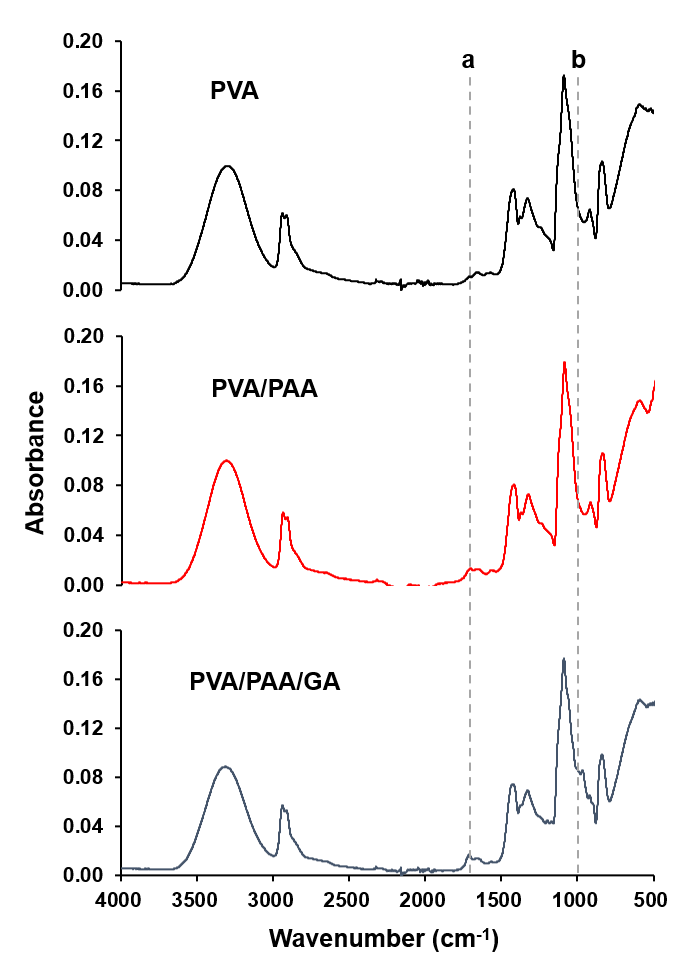

Supplement: Supplementary file 1 [file jfb-15-00262-s001.zip › Fig. S3.png]

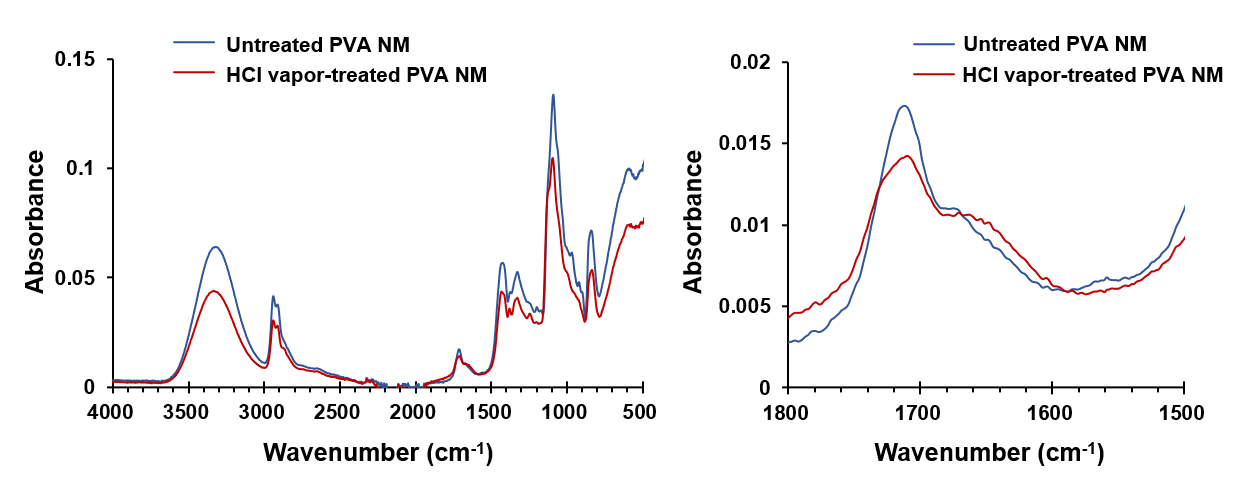

Supplement: Supplementary file 1 [file jfb-15-00262-s001.zip › Fig. S4.png]

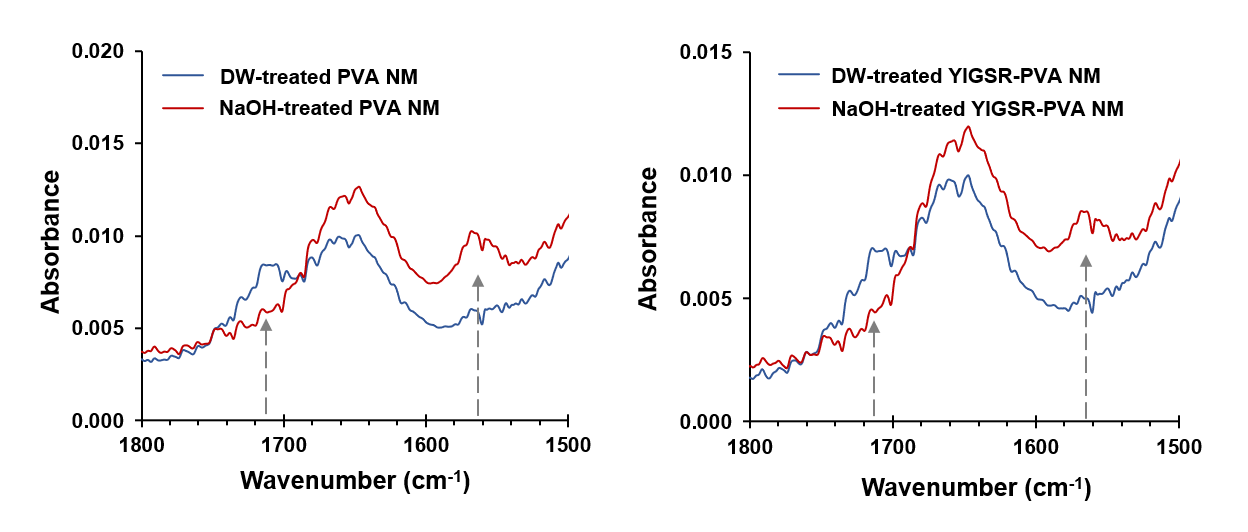

Supplement: Supplementary file 1 [file jfb-15-00262-s001.zip › Fig. S5.png]

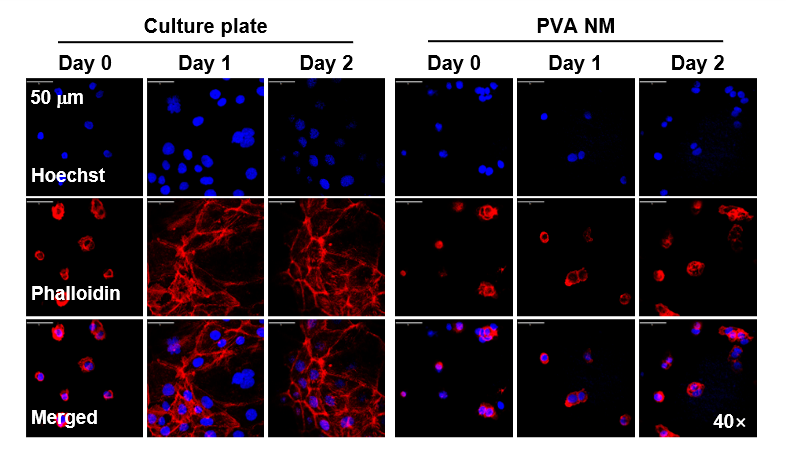

Supplement: Supplementary file 1 [file jfb-15-00262-s001.zip › Fig. S6.png]

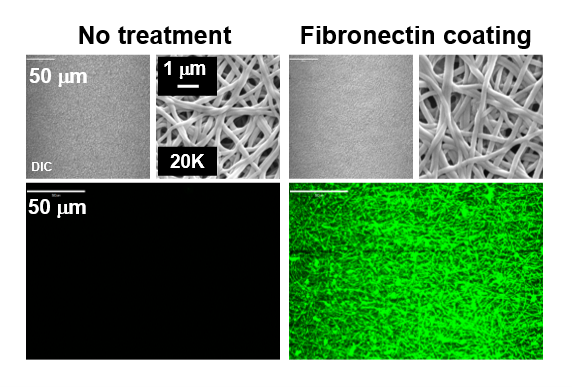

Supplement: Supplementary file 1 [file jfb-15-00262-s001.zip › Fig. S7.png]

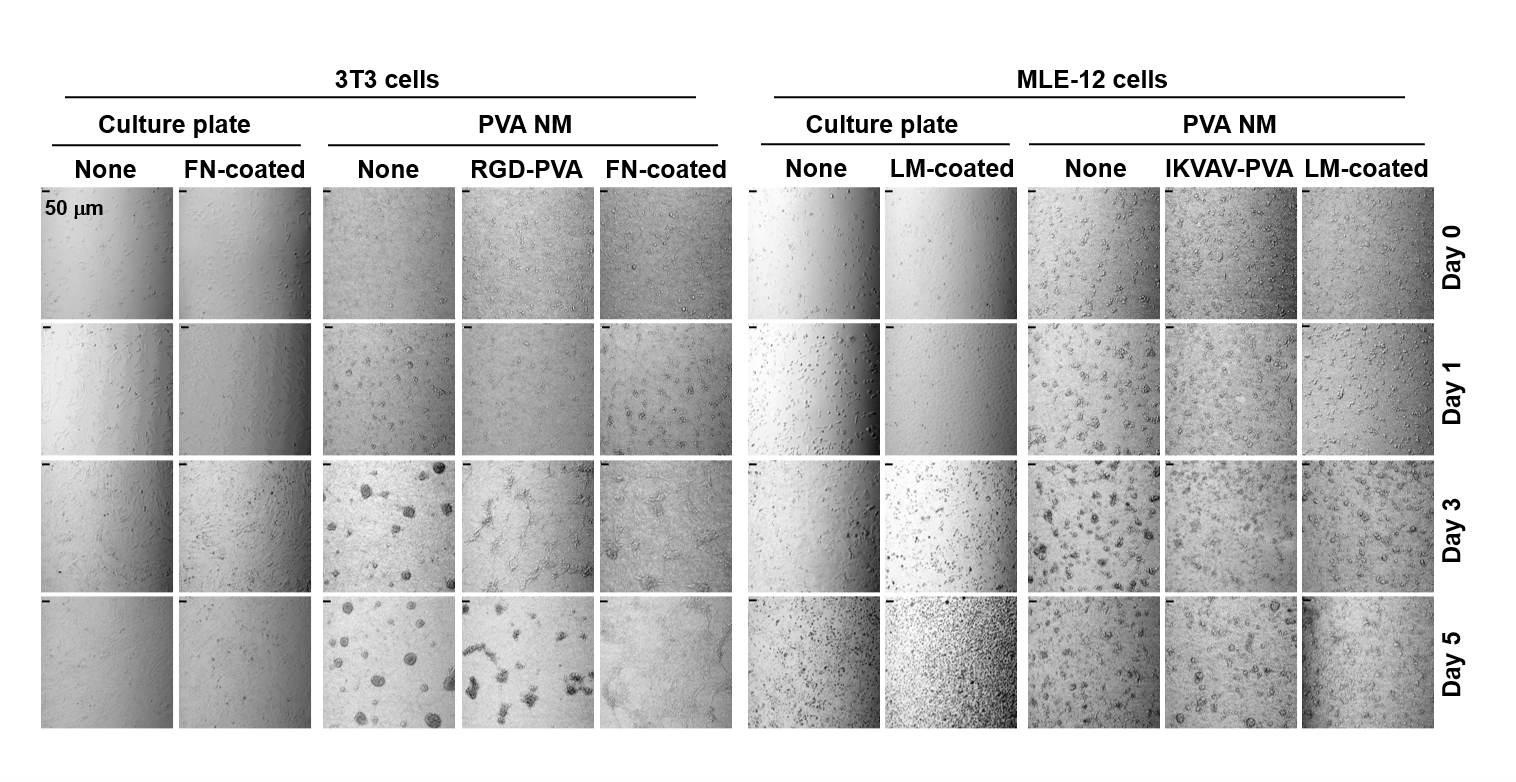

Supplement: Supplementary file 1 [file jfb-15-00262-s001.zip › Fig. S8.png]

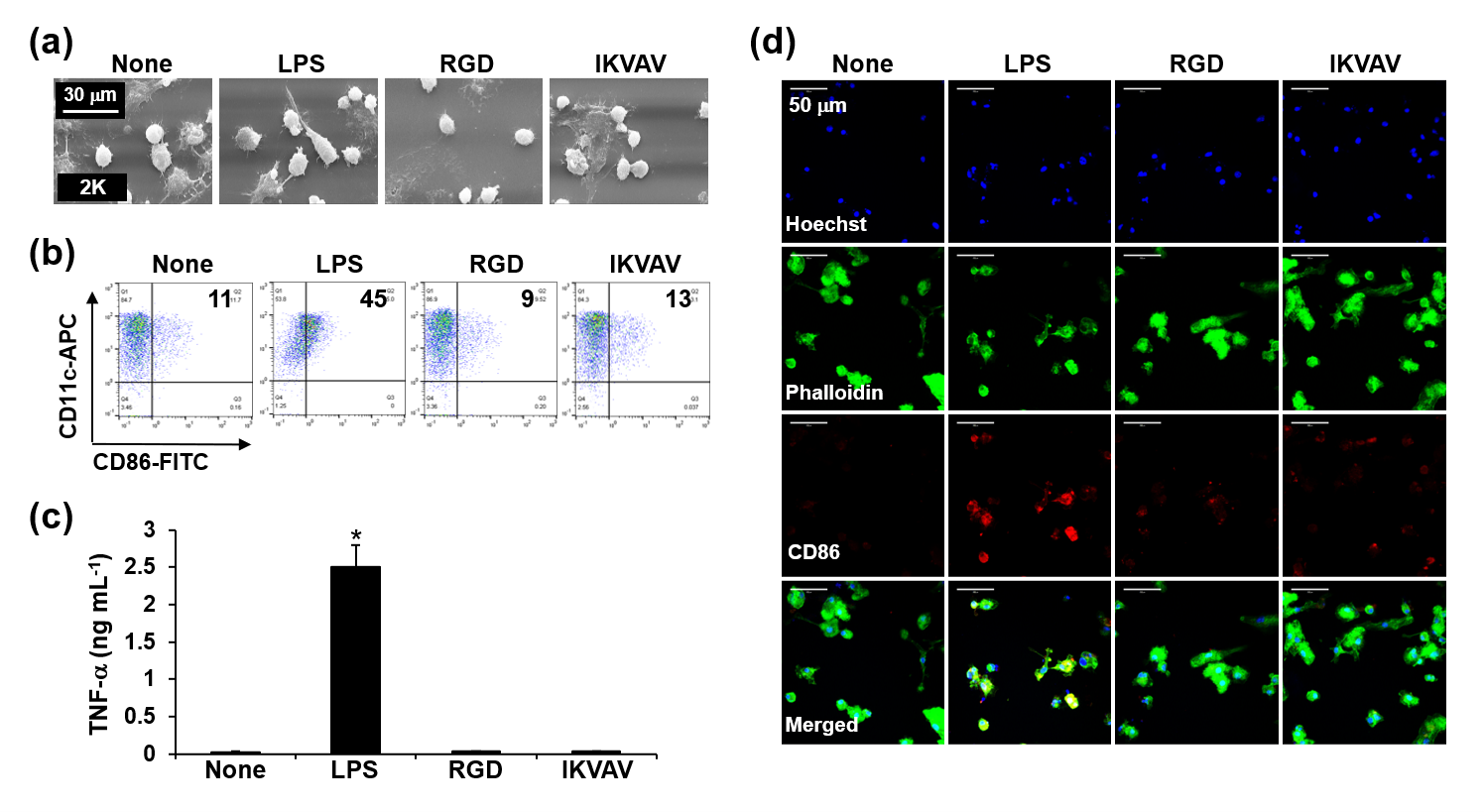

Supplement: Supplementary file 1 [file jfb-15-00262-s001.zip › Fig. S9.png]
